# *In silico* prediction of annihilators for triplet–triplet annihilation upconversion *via* auxiliary-field quantum Monte Carlo†

**DOI:** 10.1039/d0sc03381b

**Published:** 2020-11-17

**Authors:** John L. Weber, Emily M. Churchill, Steffen Jockusch, Evan J. Arthur, Andrew B. Pun, Shiwei Zhang, Richard A. Friesner, Luis M. Campos, David R. Reichman, James Shee

**Affiliations:** Department of Chemistry, Columbia University 3000 Broadway New York NY 10027 USA jshee@berkeley.edu; Schrodinger Inc 120 West 45th Street New York NY 1003 USA; Center for Computational Quantum Physics, Flatiron Institute 162 5th Avenue New York NY 10010 USA; Department of Physics, College of William and Mary Williamsburg VA 23187 USA

## Abstract

The energy of the lowest-lying triplet state (T1) relative to the ground and first-excited singlet states (S0, S1) plays a critical role in optical multiexcitonic processes of organic chromophores. Focusing on triplet–triplet annihilation (TTA) upconversion, the S0 to T1 energy gap, known as the triplet energy, is difficult to measure experimentally for most molecules of interest. *Ab initio* predictions can provide a useful alternative, however low-scaling electronic structure methods such as the Kohn–Sham and time-dependent variants of Density Functional Theory (DFT) rely heavily on the fraction of exact exchange chosen for a given functional, and tend to be unreliable when strong electronic correlation is present. Here, we use auxiliary-field quantum Monte Carlo (AFQMC), a scalable electronic structure method capable of accurately describing even strongly correlated molecules, to predict the triplet energies for a series of candidate annihilators for TTA upconversion, including 9,10 substituted anthracenes and substituted benzothiadiazole (BTD) and benzoselenodiazole (BSeD) compounds. We compare our results to predictions from a number of commonly used DFT functionals, as well as DLPNO-CCSD(T_0_), a localized approximation to coupled cluster with singles, doubles, and perturbative triples. Together with S1 estimates from absorption/emission spectra, which are well-reproduced by TD-DFT calculations employing the range-corrected hybrid functional CAM-B3LYP, we provide predictions regarding the thermodynamic feasibility of upconversion by requiring (a) the measured T1 of the sensitizer exceeds that of the calculated T1 of the candidate annihilator, and (b) twice the T1 of the annihilator exceeds its S1 energetic value. We demonstrate a successful example of *in silico* discovery of a novel annihilator, phenyl-substituted BTD, and present experimental validation *via* low temperature phosphorescence and the presence of upconverted blue light emission when coupled to a platinum octaethylporphyrin (PtOEP) sensitizer. The BTD framework thus represents a new class of annihilators for TTA upconversion. Its chemical functionalization, guided by the computational tools utilized herein, provides a promising route towards high energy (violet to near-UV) emission.

## Abstract 2

Electronic structure theories such as AFQMC can accurately predict the low-lying excited state energetics of organic chromophores involved in triplet–triplet annihilation upconversion. A novel class of benzothiadiazole annihilators is discovered.

## Graphical abstract

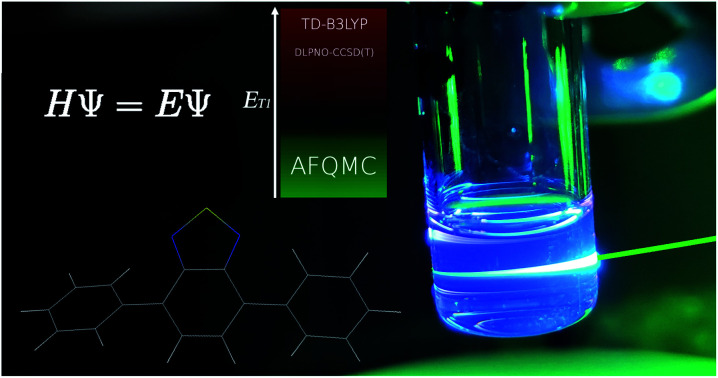

## 1 Introduction

**Fig. 1:**
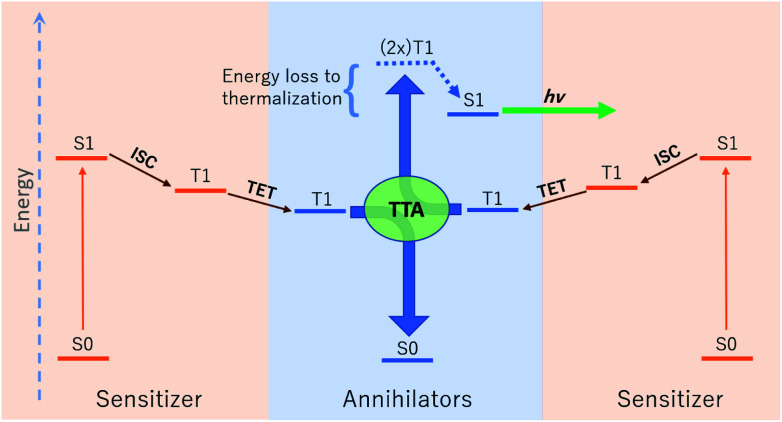
A schematic of photon upconversion *via* triplet–triplet annihilation (TTA). First, the sensitizer is photoexcited to the first excited singlet state (S1), before undergoing rapid intersystem crossing (ISC) to a long-lived triplet state. Collision with an annihilator enables transfer of the triplet state to an annihilator *via* Dexter triplet–triplet energy transfer (TET). Two annihilators in the T1 state can then undergo TTA in a spin-allowed transition resulting in one S1 and one ground state annihilator, the former of which can then emit a high energy photon *via* fluorescence. Note that in each step excess energy is lost as heat to the surroundings.

The relative energetic landscape involving states of different spin multiplicities is of essential importance in photoredox catalysis,^1–3^ the design of light emitting diodes,^4^ and optical processes such as singlet fission,^5^ thermally activated delayed fluorescence (TADF),^6^ and upconversion.^4,7^ In particular, for a system with a singlet ground state (S0), the most relevant quantities for these applications are typically the energies of the first excited singlet state (S1) and the lowest-lying triplet state (T1). Triplet–triplet annihilation (TTA) upconversion is a process which enables a system to emit photons of an energy higher than the energy of absorbed photons. This phenomenon has been used to increase the theoretical efficiency of photovoltaics,^4,8^ and to perform optogenetic manipulations and photocatalytic reactions with visible light in media (*e.g.* biological tissue) accessible only by photons of lower energy.^1,9^ A schematic of TTA upconversion is shown in Fig. 1. Following photoexcitation of a sensitizer to the S1 state, intersystem crossing (ISC) populates a relatively long-lived triplet state, T1. The sensitizer then undergoes Dexter triplet–triplet energy transfer (TET) to excite a separate molecular species, known as the annihilator, into a T1 state. Two annihilators excited to their T1 states can then undergo TTA to yield one annihilator in the S1 state and the other reverted to the ground S0 state.^10^ Thus far, there are few families of annihilators capable of emitting high energy blue to near-UV light.^4,11^ these include 9,10 substituted anthracenes,^4,11–14^*para*-terphenyl,^15^ pyrene,^16^ and 2,5-diphenyloxazole.^17,18^ Enlarging the chemical space of high energy upconverting annihilators would therefore represent a significant advancement towards the widespread use of photon upconversion for a variety of applications.

Thermodynamically, upconversion requires that (a) the sensitizer T1 energy be higher than that of the annihilator for TET, and (b) twice the annihilator T1 energy exceed the annihilator S1 energy for TTA.^19,20^ However, the degree of exothermicity for both of these processes translates directly to the amount of thermal energy lost to heat during TET and TTA, respectively. When designing optimal sensitizer/annihilator pairs to minimize energetic losses, it is important to know the relative energy levels of these excited states. For example, in addition to high fluorescence yields, TTA annihilators should exhibit a minimally positive gap between twice the T1 and S1 to reduce energy loss to thermalization. While S1 energies can be extracted from experimental spectra (*e.g. via* estimation of the energetic location of the zero-phonon line), the triplet energy can be challenging to obtain experimentally.^21–23^ The minimal (or lack of) phosphorescence is largely due to competing non-radiative pathways.

The inability to experimentally measure triplet energies has created a need which, in principle, can be met by predictions from *ab initio* computational methods. However, the development of a theoretical approach which is both accurate and feasible (with respect to computational costs) is far from trivial. The emergence of open-shell singlet ground states in large, conjugated aromatic systems reflects significant biradical, and even polyradical, character.^24^ In addition, the excited states of cyclic aromatic molecules are known to be anti-aromatic^25^ and thus similarly challenging for single-reference computational methods. These manifestations of strong electron correlation, in addition to potentially relevant phenomena such as excitations characterized by two-electron correlations and charge transfer, are well known to render commonly used computational techniques such as Kohn–Sham (KS-) or time-dependent (TD-) Density Functional Theory (DFT) unreliable.^26,27^

Several methods have been shown to be promising for the description of spin gaps of potentially biradicaloid molecules, such as spin-projected orbital-optimized MP2,^28^ spin-flip methods,^29,30^ multi-configurational pair-DFT,^31,32^ various configuration interaction approaches,^33,34^ and optimized DFT functionals.^35^ Recent efforts to reduce the scaling of Coupled Cluster (CC) methods, notably CC with singles, doubles, and perturbative triplets (CCSD(T)), have resulted in promising approaches based on domain-based localized pair natural orbital (DLPNO) approximations. Yet while these have extended the reach of CCSD(T) to larger systems,^36–38^ the potential inadequacy of the underlying theory for strong correlation still remains.^39^ Although higher order CC theories should in principle provide an increasingly accurate description, their application to relevant photoactive molecules is simply infeasible due to prohibitively high scaling with respect to system size.

We have observed that most computational results applied in the experimental literature of upconversion processes rely on TD-DFT for T1 and S1 excitation energies,^20,40–42^ despite known instabilities regarding the calculation of the T1 energy in the presence of spin-symmetry breaking.^43,44^ In this work we survey three DFT functionals prevalent in the experimental literature, including the hybrid functional B3LYP, its range-separated counterpart CAM-B3LYP,^45^ and the highly parameterized meta-GGA M06-2X.^46^ These have been shown to perform well within the Tamm-Dancoff approximation (TDA)^47^ when benchmarked against MS-CASPT2.^48^ Indeed tuning the extent of exact exchange included in hybrid DFT functionals such as these can lead to favorable cancellation of error in systems with similar charge transfer character.^48^ However, the performance of such functionals is highly variable between different families of molecules,^48–50^ complicating efforts to predict novel TTA annihilators for upconversion *a priori*.

Phaseless auxiliary-field quantum Monte Carlo (hereafter referred to as AFQMC)^51,52^ is a systematically improvable stochastic electronic structure method which scales modestly with the fourth power of the system size in our current implementation. It has recently been shown to produce accurate triplet energies for all linear polyacenes with experimentally reported T1 energies (naphthalene through pentacene) as well as for biradicals.^53^ Recent algorithmic advances^54–57^ have greatly reduced the computational costs of this methodology, enabling its use in the accurate prediction of novel chromophores, even those which may be strongly-correlated.

In this work we use AFQMC to compute T1 energies for a series of potential TTA annihilators. Anthracenes with two methyl substituents (DMA) or two phenyl substituents (DPA) are known TTA annihilators in optical upconversion schemes.^14,58^ In Section 3.1.1 we generate candidate compounds by replacing the 9,10 substituents with various functional groups that are synthetically feasible, and probe the effects, if any, on the triplet energies. We then compute the triplet energies for a series of cyano-substituted anthracenes. In Section 3.1.2 we examine derivatives of benzothiadiazole (BTD), a compound widely used in donor–acceptor paradigms typically in the context of polymers.^59,60^ It is known to have a fluorescent S1 state with an energy in the UV range (>3 eV),^61,62^ making this molecule and its derivatives potentially useful targets for TTA upconversion. We also investigate benzoselenodiazole (BSeD), which contains a selenium atom in place of sulfur. In Section 3.2, we validate the use of TD-DFT to predict adiabatic S1 energies by comparing with available experimental measurements. With an accurate computational protocol to predict both S1 and T1, we then assess the thermodynamic viability of upconversion for all molecules considered in this work by comparing twice T1 with S1. In Section 3.3 we present experimental upconversion outcomes for the phenyl-substituted BTD when coupled with platinum octaethylporphyrin (PtOEP) and zinc tetraphenylporphyrin (ZnTPP) sensitizers. This not only enables us to validate our AFQMC prediction for the triplet energy of Ph-BTD, but also provides our first example of the design of a novel, successful upconverting system informed by *ab initio* predictions. In Section 3.4 we report phosphorescence measurements of the triplet energy for the BTD series, further validating the accuracy of AFQMC for Ph-BTD and the series as a whole.

## 2 Methods

### 2.1 AFQMC methodology

AFQMC^63,64^ utilizes imaginary-time propagation to stochastically sample properties associated with a given Hamiltonian *via* a random walk within the complex manifold of Slater determinants. The exponentially growing noise that would otherwise be incurred while averaging observables in imaginary-time is controlled by the use of a trial wavefunction to implement the phaseless constraint, at the expense of a bias which can be systematically reduced *via* improvement of the trial wavefunction. The lowest-energy state of each irreducible representation of the symmetry group of the Hamiltonian can be computed by AFQMC in the same manner as the ground state (which is a special example of such a state). Our singlet calculations have *N*_*α*_ = *N*_*β*_ and triplet calculations have *N*_*α*_ = *N*_*β*_ + 2. Properties of low-lying excited states belonging to the same irreducible representation can be obtained from the AFQMC methodology *via* the use of a trial wavefunction chosen such that it is orthogonal to eigenstates of lower-energy.^65,66^ In practice, of course, the exact targeted eigenstate is unknown beforehand, necessitating the use of approximate wavefunctions obtained from other quantum chemical methods, which are typically nearly orthogonal to the ground-state. A spin filtration technique^67^ allows us to preserve the total spin (〈*S*^2^〉 = 0 and 2 for singlets and triplets, respectively) in the AFQMC projection. The use of trial wave functions which preserve or better approximate symmetries helps to improve results, as further discussed below.

The use of unrestricted single determinant trials has been shown to yield sub-kcal mol^−1^ accuracy for the triplet energies of polyacenes with closed-shell ground-states, and many biradicaloid molecules with open-shell singlet states that can be qualitatively described by two determinants.^53,68^ However, some highly multi-reference systems such as transition metal compounds require the use of non-orthogonal determinant expansions^69^ or truncated CASSCF trial wavefunctions^55,70,71^ to yield high accuracy. In this work, all AFQMC calculations implement unrestricted single-determinant trial wavefunctions selected according to the AFQMC/U protocol,^53^ except for those on the BTD and BSeD derivatives, which were found to exhibit signs of strong correlation (*vide infra*) and thus required truncated CASSCF trials. As the lowest excited states for such conjugated molecules are π to π* transitions,^72^ we use active spaces spanned by all valence π-orbitals. In the case of phenyl-substituted BTD/BSeD, the resulting active spaces were intractable, and so the three highest and lowest virtual and occupied orbitals, respectively, were neglected in active space optimization. Trial wavefunctions and all required integrals for AFQMC calculations were obtained using PySCF.^73^ Extrapolations to the complete basis set (CBS) limit were performed using DLPNO-CCSD(T) values in TZ and QZ dunning basis sets^74,75^ (see ref. 70 for details of this protocol), and dielectric solvation corrections were computed using a simple conductor-like polarizable continuum model (CPCM) at the B3LYP/TZ level. Further details regarding the AFQMC calculations can be found in the ESI.†

### 2.2 DFT and DLPNO-CCSD(T) calculations

KS-B3LYP, TD-DFT, and DLPNO-CCSD(T)^76,77^ calculations were performed with the ORCA quantum chemistry program.^78^ S0 and T1 geometries were optimized at the KS-B3LYP/cc-pVTZ level of theory. The reference wavefunctions for DLPNO-CCSD(T) calculations were chosen as follows. As large deviations from the exact *S*^2^ values were found for both S0 and T1 states of the anthracene derivatives at the UHF level, inconsistent with the stable closed-shell nature of acenes of this length,^79^ we utilize restricted orbitals for the anthracene derivatives (RHF/ROHF for S0/T1). For the BTD series, we use UHF reference wavefunctions. The semi-canonical approximation to the triples correction, DLPNO-CCSD(T_0_), was used.^77^ Henceforth, DLPNO-CCSD(T) will be refer to DLPNO-CCSD(T_0_). The “NormalPNO” cutoff was used for all DLPNO-CCSD(T) calculations.^77^

For TD-DFT calculations of adiabatic S1 energies, we correct the vertical excitation energy (with respect to S0 geometries) with a relaxation term, obtained from geometries which reflect the minimum energy of the target excited state within the TDA approximation. Subsequent single-point excitation energies were then computed without the TDA approximation. Regarding T1 calculations *via* TD-DFT, it has been found that triplet instabilities can lead to an unphysical underestimation of T1 energies especially when using functionals with a significant percentage of exact exchange, and that employing TDA can help to ameliorate this error.^43^ Since in the anthracene set all molecules exhibit notable spin contamination in the singlet state, we utilize the TDA approximation when calculating the triplet energy using TD-DFT, specifically when calculating the vertical excitation energy corresponding to the optimized geometry of S0. To report adiabatic T1 energies, we correct the vertical excitation energy with T1 geometry relaxation energies, obtained by adding the difference in total KS-DFT/B3LYP T1 energies between the optimized S0 and T1 geometries to the vertical excitation energies.

For a subset of molecules in Section 3.1.1 we investigated the importance of supplementing gas-phase electronic energy gaps with vibrational and solvation effects. We found (Table S†) that inclusion of the above effects did not change the calculated triplet energies by an amount larger than the statistical error bars of AFQMC, and thus while our results for the anthracene derivatives in this paper reflect gas-phase electronic gaps, we expect these to be close to what would be realistically measured in toluene solvent. For the BTD series, in particular MeO-BTD, which exhibits strong charge transfer characteristics, the dielectric solvation corrections were not negligible, and so our calculated values reflect a correction term obtained from separate calculations employing the CPCM continuum solvation model. All calculations for the anthracenes use the cc-pVTZ basis set, as it was found in every case to be near the complete basis set (CBS) limit. The calculated triplet energies for the BTD and BSeD series have been extrapolated to the CBS limit (using *X* = T,Q basis sets, and a 1/*X*^3^ form for the correlation energy). For the selenium complexes we use the cc-pVXZ-dkh basis set and the x1c formalism to include scalar relativistic effects. We refer the reader to the ESI† for further information.

### 2.3 Experimental methods

Details for the synthesis of Ph-BTD can be found in Section S1 of the ESI.† All starting materials were obtained from commercial sources, including Fisher Scientific, TCI Chemical, and Strem Chemicals. BTD (ACROS Organics), ZnTPP (Fisher Scientific), and PtOEP (Sigma-Aldrich) were purchased and used without further purification.

NMR spectra were collected on a Bruker 500 MHz spectrometer at ambient temperature. UV-Vis absorption spectra were collected by a Technologies Cary 60 UV-Vis spectrophotometer. Steady-state photoluminescence spectra were collected by an Ocean Optics QEPro spectrometer.

Solution concentrations for photon upconversion studies were prepared as 1 × 10^−5^ M sensitizer and 1 × 10^−3^ M annihilator in degassed anhydrous toluene. Solutions for each sensitizer–annihilator pair were made in a nitrogen glovebox, sealed, and removed from the glovebox for upconversion photoluminescence study.

Phosphorescence measurements were taken at 77 K in a frozen solution of methylcyclohexane (BTD/CN-BTD) and methylcyclohexane/iodomethane (2 : 1 v/v) (MeO-BTD, Ph-BTD) (details in ESI†).

## 3 Results

### 3.1 Calculating accurate triplet energies for TTA upconversion annihilators

#### 3.1.1 Anthracene 9,10 functionalization

As a preliminary test, to investigate the accuracy of unrestricted single-determinant trials for substituted acenes, we compared AFQMC/UHF and AFQMC/UB3LYP with AFQMC/CAS for benzonitrile, a small but representative system for which large CASSCF trial wavefuctions (and thus near exact AFQMC energies) can readily be obtained. In previous studies we have shown that using such trial wavefunctions can largely eliminate the bias from the phaseless constraint such that the resulting predictions agree well with experimental measurements.^53,55,70,80^ For benzonitrile we use an active space of 8 electrons in 16 orbitals (8e16o), representing the full π system plus a second set of virtual orbitals. The results are shown in Table 1. While all methods produce triplet energies above the lower bound from experiment, the result from the KS-B3LYP trial is within 0.01 eV of that from the CASSCF trial. This is consistent with our previous validation of the AFQMC/U protocol for small-molecule biradicals and unsubstituted acenes, in which UHF is used as a trial unless there is significant spin contamination (in the case of benzonitrile singlet, where 〈*S*^2^〉 = 0.59), in which case an unrestricted Kohn–Sham (UKS) trial is used.^53^ A similar protocol has been shown to improve the accuracy of CC methods.^81^

**Table 1:** AFQMC results from various trial wavefunctions for the adiabatic triplet energy of benzonitrile, in eV. Parentheses denote statistical error of AFQMC, *i.e.* 3.61(6) denotes 3.61 ± 0.06

AFQMC/UHF	AFQMC/UKS	AFQMC/CAS	Expt.^82^
3.86(9)	3.62(8)	3.61(7)	>3.35

**Fig. 2:**
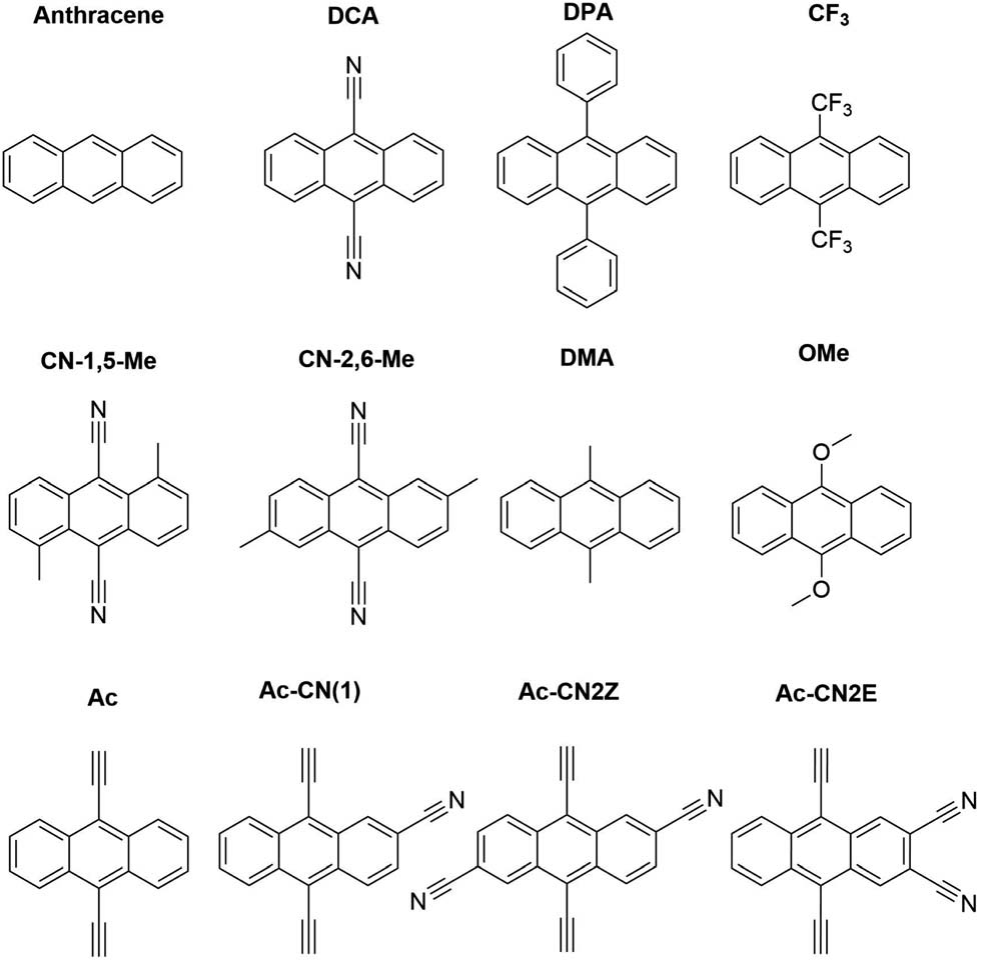
Anthracene derivatives included in this study.

**Fig. 3:**
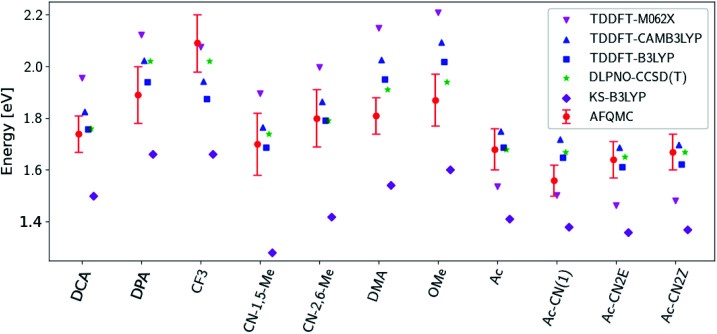
A comparison of T1 values for included TD-DFT functionals, KS-B3LYP, and DLPNO-CCSD(T). Note that KS-B3LYP obtains significantly lower triplet energies when compared to AFQMC for the entire series, whereas DLPNO-CCSD(T) is within one standard deviation from AFQMC for the majority of the compounds involved, with a mean average deviation from AFQMC below the average statistical error of the latter. All DLPNO-CCSD(T) and AFQMC values reflect gas-phase calculations in the cc-pVTZ basis. Numbers for these, as well as CBS/solvation corrections, can be found in the ESI.†

Fig. 3 presents adiabatic triplet energies obtained from KS-DFT, TD-DFT with three different representative functionals, DLPNO-CCSD(T), and AFQMC/U for the functionalized anthracenes shown in Fig. 2, along with mean absolute deviations (MADs) between each method and AFQMC shown in Table 2. In nearly every case, the DLPNO-CCSD(T) and AFQMC/U results agree to within the statistical error bars of the latter, with the MAD between AFQMC/U and DLPNO-CCSD(T) (0.05 eV) being less than the mean statistical error from AFQMC/U (0.09 eV).

**Table 2:** A comparison in eV of DLPNO-CCSD(T), KS-DFT, and TD-DFT results for T1 energies of anthracene derivatives, including mean absolute deviation (MAD), mean signed deviation (MSD), and maximum deviation (Max) *versus* AFQMC/U. Both DLPNO-CCSD(T) and TD-B3LYP have an MAD below the average statistical error of AFQMC (0.09 eV), although TD-B3LYP exhibits a higher maximum deviation of 0.215 eV *vs.* AFQMC

	KS-B3LYP	TD-B3LYP	TD-CAM-B3LYP	TD-M062X	DLPNO-CCSD(T)
MAD *vs.* AFQMC	0.297	0.070	0.112	0.191	0.051
MSD *vs.* AFQMC	−0.297	0.013	0.085	0.085	0.037
Max *vs.* AFQMC	0.430	0.215	0.223	0.338	0.135

We are aware of only one direct experimental measurement of the triplet energy in a comparable solvent for this set of molecules, namely for DCA in toluene, which has a value of 1.8 eV.^83^ Both DLPNO-CCSD(T) and AFQMC/U are in good agreement with this value, whereas KS-DFT with the B3LYP functional systematically underestimates the gap. In addition, we previously reported an AFQMC/U value for anthracene within 0.04 ± 0.05 eV of a gas phase experimental measurement.^53,79^ Recently, DPA was reported to have a triplet energy of about ≃1.75 eV, measured in a polymer host matrix consisting of poly(4-bromostyrene) and benzophenone.^84^ Neglecting the experimental uncertainty (which was not reported), this is slightly outside of the error bars of our AFQMC calculation (1.89 ± 0.11 eV). We postulate that this possible, small discrepancy is due to the environment of the experiment. We also note that, among the theoretical methods considered (barring KS-DFT/B3LYP, which underestimates the triplet energy of DCA by ≃0.3 eV), AFQMC yields the closest value to experiment. These available comparisons suggest that AFQMC provides reliable predictive power for this class of anthracene derivatives.

#### 3.1.2 BTD/BSeD based TTA annihilators

**Fig. 4:**
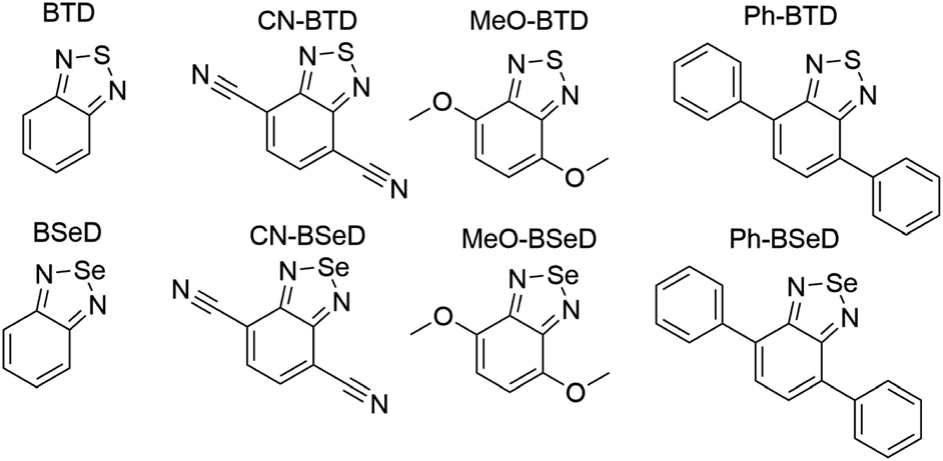
BTD and BSeD derivatives included in this study.

In this section we investigate the triplet energies of a set of synthetically-feasible derivatives of benzothiadiazole (BTD) and benzoselenodiazole (BSeD), shown in Fig. 4. We find that these molecules exhibit a substantial degree of electron correlation, *e.g.* the CASSCF wavefunctions for S0 and T1 of Ph-BTD contain roughly 40k and 60k determinants, respectively (representing 99.5% of the sum of squares of CI coefficients). In this regime, AFQMC/UKS is no longer expected to produce accurate results (indeed, AFQMC/UKS and AFQMC/CAS produced results differing by 0.26 ± 0.08 eV for BTD); we therefore use AFQMC/CAS. It is known that initializing CASSCF active spaces with the full π system, as identified visually at the restricted HF level, is necessary for quantitative results in conjugated aromatics.^72^ We follow this protocol for all systems except those with phenyl groups, in which case we had to exclude the lowest three occupied orbitals and highest three virtuals from the active space due to computational limitations. Due to the large computational cost of these calculations, only the first ∼500 determinants were maintained in the CASSCF trials, which still represented over 94% of the CI weights for each molecule.

Whereas the anthracenes exhibit negligible basis set incompleteness effects (Table S1†), this is not the case for the BTDs and BSeDs, and so all AFQMC and DLPNO-CCSD(T) numbers for these molecules reflect an extrapolation to the CBS limit. Additionally, the relatively more substantial charge-transfer character in some cases, *vs.* the anthracenes, can lead to a significant solvent correction, *e.g.* a shift of −0.15 eV for MeO-BTD. For consistency, we therefore include the correction from the dielectric continuum model for all BTD and BSeD derivatives.

**Fig. 5:**
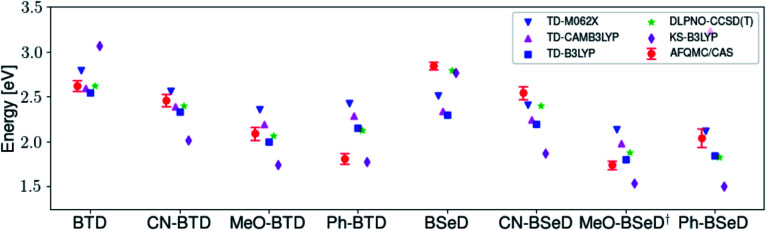
A comparison of the triplet energies calculated with TD-DFT, KS-B3LYP, and DLPNO-CCSD(T). Once more KS-B3LYP obtains significantly lower triplet energies as compared to AFQMC for nearly all species, except notably for the parent BTD and BSeD compounds, and Ph-BTD. Ph-BTD stands out as an outlier, with the maximum deviation of DLPNO-CCSD(T) from AFQMC at ∼0.3 eV. Numbers can be seen in the ESI.† MeO-BSeD represents AFQMC in the triple-ζ basis (*i.e.* not CBS limit).

While for single-reference systems, *i.e.* those that can be well-described by one orbital-occupancy configuration, DFT and CCSD(T) methods are capable of producing robust accuracy, we can be less confident that these methods will produce accurate T1 energies for the BTD and BSeD derivatives. Interestingly, we find good agreement between DLPNO-CCSD(T) and AFQMC, excepting the case of Ph-BTD (Table 3). Screening for spin contamination in the stable UHF references revealed minimal spin contamination for all triplet species (except Ph-BTD), and significant deviations from the exact value (0) for all singlets. This implies that states of different spin-multiplicities (*e.g.*, singlet, triplet, quintet) are sufficiently close in energy that they “mix” to lower the energy at the mean-field level (at the expense of spin symmetry breaking). The determinant constructed from unrestricted Kohn–Sham (UKS) orbitals removed the spin contamination, and using this as a reference wavefunction resulted in a nearly equivalent DLPNO-CCSD(T)/cc-pVTZ result for all species, suggesting that the use of spin-contaminated reference orbitals cannot account for the deviation from the AFQMC result. In Section 3.3 we will show experimental evidence which suggests that the triplet energy as predicted by AFQMC/CAS is accurate. TD-DFT with the B3LYP functional performs best (with respect to AFQMC) among the DFT methods investigated, while inclusion of long-range HF exchange with the CAM-B3LYP functional worsens the MAD by more than a factor of two. A plot of the calculated triplet energies for AFQMC/CAS and alternate methods can be seen in Fig. 5. It should be noted that all methods follow the same general trend, where the triplet energy of BTD > CN-BTD > MeO-BTD > Ph-BTD, consistent with S1 calculations that are presented and rationalized based on π-system extension and donor–acceptor paradigms in Table S8.†

**Table 3:** A comparison in eV of DLPNO-CCSD(T), KS-DFT, and TD-DFT results for T1 energies of a set of substituted BTD and BSeD compounds benchmarked against AFQMC/CAS, including mean absolute deviation (MAD), mean signed deviation (MSD), and maximum deviation (Max) *versus* AFQMC/CAS. All methods have significantly higher maximum deviations from AFQMC than was found for the anthracenes, as is expected given the larger degree of electron correlation observed in these compounds. DLPNO-CCSD(T) and TD-B3LYP again have the lowest and second lowest MADs *vs.* AFQMC, respectively

Species	KS-B3LYP	B3LYP	CAM-B3LYP	M062X	DLPNO-CCSD(T)
MAD *vs.* AFQMC/CAS	0.35	0.22	0.36	0.26	0.12
MSD *vs.* AFQMC/CAS	−0.23	−0.12	0.14	0.15	0.00
Max *vs.* AFQMC/CAS	0.67	0.55	1.20	0.62	0.32

### 3.2 Predicting upconversion activity

#### 3.2.1 S1 energies

In contrast to T1, S1 can readily be measured experimentally. However, in order to make predictions about the thermodynamics of new potentially upconverting systems, it is necessary to accurately calculate S1. Previous studies have shown that the CAM-B3LYP functional yields S1 energies of extended polyaromatics that are very close to experimental measurements.^43,45,87^ In Table 4 we have collected a set of conjugated potential annihilators for which experimental S1 energies are available. This set supplements the molecules in this study with 7 tetracene derivatives containing 0–4 cyano substituents. The mean absolute error (MAE) with respect to experiment is 0.056 eV, giving us confidence that this functional can be used to compute S1 energies for these molecules with sufficient accuracy (*i.e.* comparable to the statistical error bars on our AFQMC T1 calculations). We note that it is possible to obtain S1 energies with AFQMC *via* an appropriately imposed symmetry constraint, and present an example computing S1 and T1 for anthracene in the ESI.† Given the demonstrated accuracy of TD-DFT methods, we leave this for future work.

**Table 4:** CAM-B3LYP TD-DFT results in eV for S1 energies of all molecules, including a subset of CN substituted tetracenes, with available experiments. The MAE was found to be 0.056 eV with respect to available experiments. Structures for the tetracenes can be found in the ESI

Species	S1 (TD-DFT)	Expt	Difference
**Tetracenes**			
CN0	2.30	2.30	0.00
CN1	2.28	2.26	0.02
CN2T	2.25	2.21	0.04
CN2E *cis*	2.25	2.23	0.02
CN2H	2.19	2.16	0.03
CN3	2.23	2.20	0.03
CN4	2.21	2.19	0.02

**Anthracenes**			
DPA	3.14		
DMA	3.10		
OMe	3.06		
CF3	3.03		
CN-2,6-Me	2.93		
DCA	2.92	2.90 (ref. 83)	0.02
CN-1,5-Me	2.79		
Ac	2.88	2.80	0.08
Ac-CN1	2.82	2.71	0.11
Ac-CN2E	2.78	2.66	0.12
Ac-CN2Z	2.77	2.65	0.12

**BTD derivatives**			
BTD	3.86	<3.97 (ref. 62)	<0.11
CN-BTD	3.64		
MeO-BTD	3.05	3.13 (ref. 85)	0.08
Ph-BTD	3.04	3.08 (ref. 86)	0.04
BSeD	3.57		
CN-BSeD	3.41		
MeO-BSeD	2.78		
Ph-BSeD	2.85		

#### 3.2.2 Energetic efficiency of TTA upconverting candidates

**Fig. 6:**
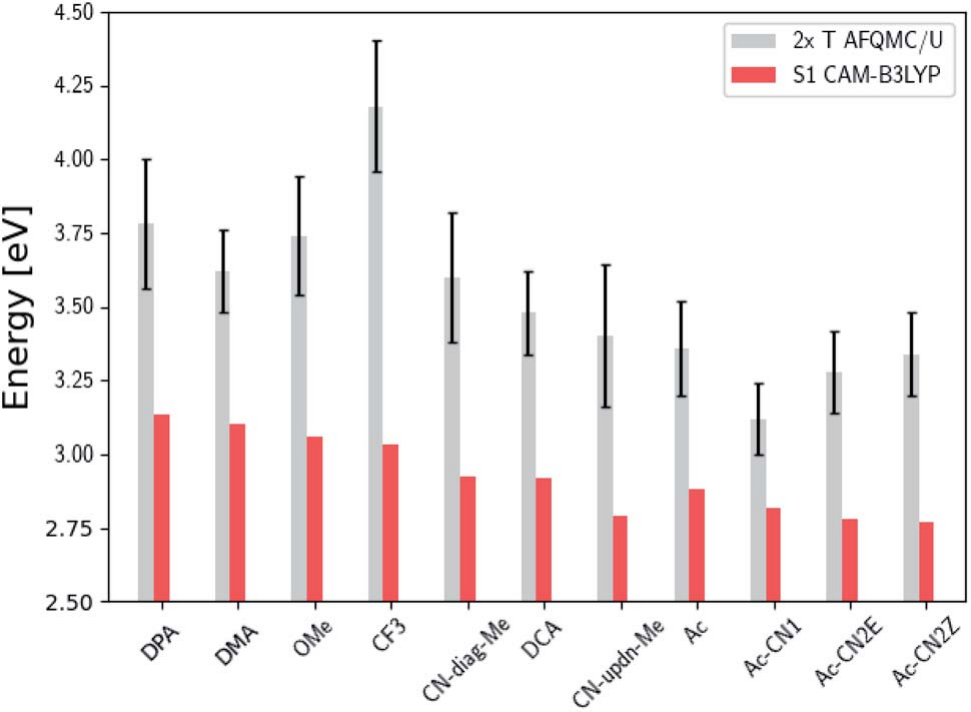
Comparison of the predicted gas phase 2× T1 energetic values, as calculated using AFQMC/U, with S1 values obtained from TD-DFT/CAM-B3LYP for the anthracene derivatives. Note that the mono-substituted CN-anthracene, Ac-CN1, exhibits the lowest difference between 2× T1 and S1, and therefore the lowest potential energy loss during TTA. Further note the destabilization of the triplet state for the highly inductively-withdrawing CF_3_ substituted species.

**Fig. 7:**
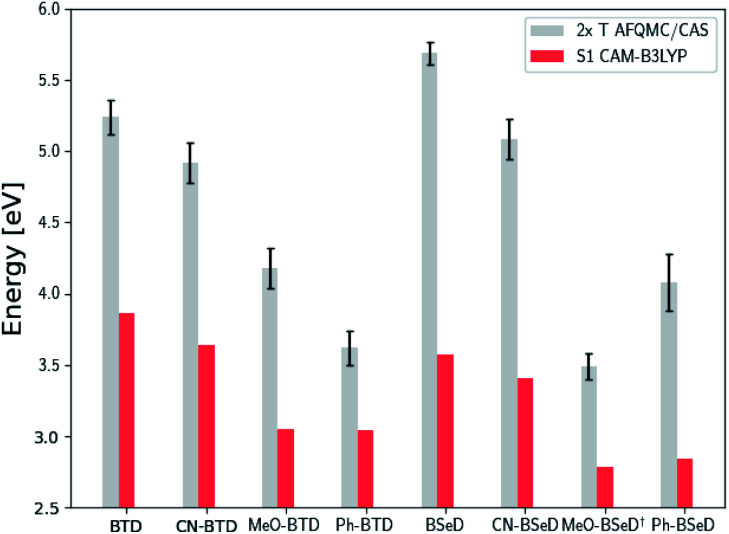
Comparison of the predicted gas-phase 2× T1 energetic values as calculated using AFQMC/CAS, *versus* S1 values obtained from TD-DFT/CAM-B3LYP for the BTD/BSeD derivatives. All molecules exhibit 2× T1 > S1, and therefore are exothermic towards upconversion. Substitution on the phenyl ring of BTD leads to a lowering of both the singlet and triplet excited states in all cases, and to a larger extent for the electron donating functional groups. Ph-BTD exhibits the lowest 2× T1 − S1.† MeO-BSeD represents AFQMC in the triple-ζ basis (*i.e.* not CBS limit).

While the inequality 2× T1 > S1 is a thermodynamic prerequisite for upconversion, achieving efficiencies necessary for practical applications may require additional considerations. For example, it is often preferable to minimize the energy loss during TTA by engineering 2× T1 − S1 to be minimally positive.^12^ In Fig. 6 and 7 we compare 2× T1, as predicted *via* AFQMC, with S1, as predicted from TD-DFT/CAM-B3LYP. Among the anthracene derivatives, Ac-CN1 is predicted to be the most efficient annihilator by this metric. Among the BTD and BSeD compounds, 2× T1 − S1 is smallest for Ph-BTD.

We note in passing that the trends in the S1 gaps for the molecules shown in Fig. 6 and 7 can be qualitatively predicted by simple models that describe extended conjugated molecules, *e.g.* particle-in-a-box π-extension and donor–acceptor (charge transfer) paradigms. A discussion rationalizing S1 energies in these molecules is presented in the ESI.† Similar trends in triplet energies are found, albeit with notable outliers, such as CF_3_-anthracene and Ac-CN1. These observations, particularly the discrepancies between trends in S1 and T1, further emphasize the need for quantitatively accurate *ab initio* electronic structure methods for the calculation of triplet energies.

### 3.3 Observation of upconversion

Two observations motivated us to experimentally investigate Ph-BTD. First, the predicted triplet energies for Ph-BTD *via* all TD-DFT methods and DLPNO-CCSD(T) are significantly larger than that predicted by AFQMC/CAS, by 0.3–0.67 eV, representing a significant discrepancy between traditional electronic structure methods and AFQMC. Second, of the BTD and BSeD series, Ph-BTD is predicted (by AFQMC) to have the smallest energetic loss from TTA. We therefore decided to experimentally test for upconversion activity by coupling the Ph-BTD annihilator with two different sensitizers, platinum octaethylporphyrin (PtOEP) and zinc tetraphenylporphyrin (ZnTPP), with known experimental triplet energies of 1.91 eV (ref. 88) and 1.61 eV,^89^ respectively.

**Fig. 8:**
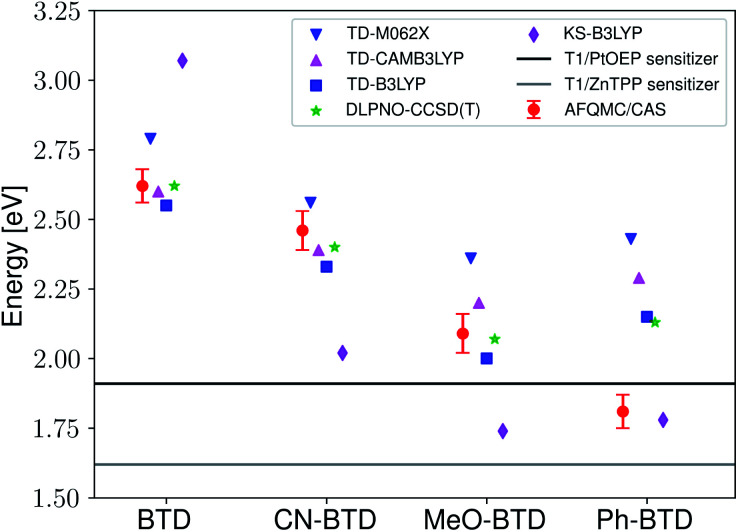
Predicted AFQMC values *versus* experimental sensitizer triplet energies for the BTD series. The sensitizer triplet energy is generally required to be above that of the annihilator in order for efficient, exergonic triplet–triplet energy transfer to occur, and so we expect Ph-BTD to upconvert when paired with PtOEP.

In upconverting systems, the initial population of a sensitizer's S1 state *via* photoexcitation is followed by ISC to the sensitizer's T1 state, and then by TET, in which the energy of the T1 state of the sensitizer is transferred to form the T1 state of the annihilator (Fig. 1). TET is thermodynamically allowed when the triplet energy of the annihilator is downhill from that of the sensitizer. As can be seen in Fig. 8, the PtOEP and ZnTPP sensitizer triplet energies effectively sandwich our AFQMC-predicted triplet energy for the Ph-BTD annihilator, 1.77(6) eV. We can thus expect that if our AFQMC prediction is correct, the PtOEP/Ph-BTD system should be able to upconvert, whereas the ZnTPP/Ph-BTD system should not.

**Fig. 9:**
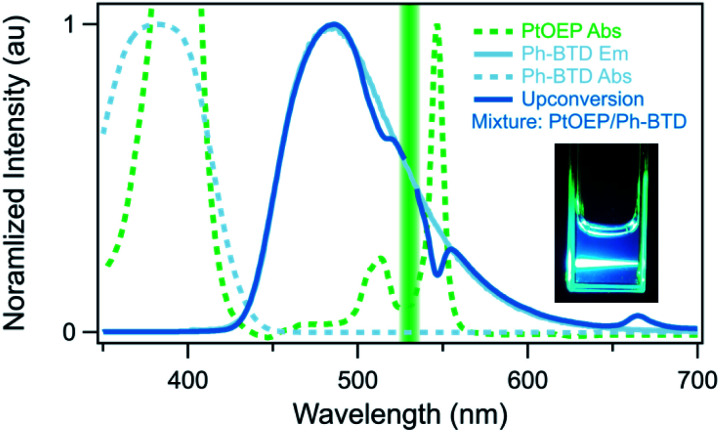
Photoluminescence confirmation of upconversion (dark blue) by the Ph-BTD/PtOEP system in toluene upon excitation with 532 nm light (green line), absorption of PtOEP (green dashed), absorption (light blue dashed) and photoluminescence (light blue solid) spectra of Ph-BTD. Note that the Ph-BTD/PtOEP upconversion system emits at a higher energy than the excitation wavelength and that the Ph-BTD does not directly absorb light at the excitation wavelength. Visual observation (insert) corroborates this measurement.

**Fig. 10:**
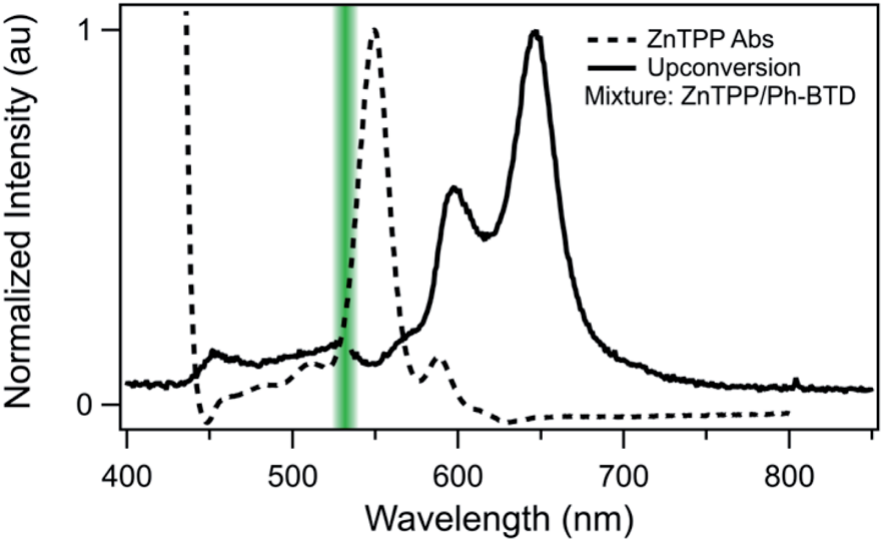
Absorption (dashed) spectrum of ZnTPP, and photoluminescence (solid) of the Ph-BTD/ZnTPP pair in toluene with excitation at 532 nm (green line). The emission of the mixture matches that of ZnTPP (Fig. S2†), signifying inefficient TET to Ph-BTD, as predicted by the relative T1 energy levels.

Indeed, Ph-BTD exhibits the ability to upconvert when coupled to a PtOEP sensitizer, with an anti-Stokes shift of approximately 0.2 eV from the excitation energy to the peak emission of the system, as seen in Fig. 9. This provides evidence for the triplet energy of Ph-BTD being below 1.91 eV, consistent with our AFQMC predictions. Note that none of the TD-DFT results are consistent with this observation, and neither is DLPNO-CCSD(T). KS-DFT with the B3LYP functional is consistent with this observation, but as it underestimates triplet energies for most compounds it is most probable that this agreement is fortuitous. On the other hand, the mixture of ZnTPP and Ph-BTD shows phosphorescence of the sensitizer (Fig. 10), indicating that prominent upconversion does not occur, thus supporting our prediction that the triplet energy of Ph-BTD is too large for effective TET from ZnTPP. These two experimental observations imply that 1.61 eV < Ph-BTD(T1) < 1.91 eV, consistent with our AFQMC/CAS prediction.

We comment that endothermic entropically-driven, endothermic TET has previously been reported in the literature.^9,90,91^ In these representative instances, TET is found to be up-hill by <0.1 eV, which is a significantly smaller number than the smallest TDDFT-predicted endothermicity of PtOEP/Ph-BTD, which is 0.25 eV (B3LYP). This very extreme endothermicity, in light of literature precedent, most likely would not allow forward TET. This strengthens the claim that these TDDFT and DLPNO-CCSD(T) methods are unreliable in predicting the triplet energy of this PtOEP/Ph-BTD system.

### 3.4 Comparison to low temperature phosphorescence

We performed low-temperature phosphorescence for the series of functionalized BTD compounds at 77 K frozen in methyl-cyclohexane (BTD, CN-BTD) and a 1 : 2 wt. mixture of iodomethane and methyl-cyclohexane (MeO-BTD, Ph-BTD). The resulting spectra can be seen in the ESI,† and the estimated 0–0 triplet energies are shown in Table 5. Importantly, AFQMC/C calculations in the CBS limit and with implicit solvent corrections agree with experimental triplet energies to within 0.11 ± 0.07 eV for MeO-BTD and to within 0.04 ± 0.06 eV for Ph-BTD. The experimental triplet energies of BTD and CN-BTD are reported to be ≃0.28 and ≃0.38 eV below that of the AFQMC-predicted values; in general, none of the electronic structure predictions were consistent with these experiments, though KS-B3LYP was within 0.1 eV for CN-BTD (it should be noted that KS-B3LYP was off by ≃0.7 eV for BTD).

**Table 5:** Experimental (phosphorescence) estimations of the 0–0 triplet energy, compared against computational predictions for the monomer (CBS with dielectric solvation corrections). BTD is severely overestimated by all methods, whereas CN-BTD is overestimated by all but KS-B3LYP. In the case of Ph-BTD, AFQMC/C agrees to within statistical error, and indeed exhibits the lowest deviation from experiment, closely followed by KS-B3LYP

Species	Experiment	AFQMC/C	KS-B3LYP	B3LYP	CAM-B3LYP	M062X	DLPNO-CCSD(T)
BTD	2.33	2.62(6)	3.07	2.55	2.60	2.79	2.62
CN-BTD	2.08	2.46(7)	2.02	2.33	2.39	2.56	2.40
MeO-BTD	1.98	2.09(7)	1.74	2.00	2.20	2.36	2.07
Ph-BTD	1.85	1.81(6)	1.78	2.15	2.29	2.43	2.13

Due to the significant discrepancy between all computational methods and experiment for BTD and CN-BTD, we attempted to pinpoint global sources of computational error. A literature search revealed that there is some precedence for favorable anti-square dimerization of benzothiadiazoles.^92^ To explore this, we estimated dimerization free energies at the ωB97X-V/cc-pVTZ-DK level of theory, which suggest that both BTD and CN-BTD exist as gas phase dimers at 77 K. Additionally, DLPNO-CCSD(T)/cc-pVTZ calculations of the dimers effectively reduce the error for BTD and CN-BTD from 0.29 eV and 0.32 eV to 0.09 eV and 0.21 eV (see ESI† for further details).

## 4 Discussion

The results of this study serve as a caution to practitioners relying on DFT methods to predict triplet energies of various types of molecules, especially in the absence of careful, system-specific benchmarking, despite the convenience resulting from the speed, black-box nature, and frequent accuracy of such calculations. The data suggest that, of the DFT-based approaches, TD-B3LYP shows the highest level of accuracy with respect to AFQMC reference values, and on average its predictions lie within the statistical error bars of the AFQMC calculations for the weakly correlated anthracene derivatives. This is consistent with ref. 50, which found similar accuracy for a set of annihilators including diphenyl anthracene. The so-called “gold standard” of traditional electronic structure theory, CCSD(T), here represented by the DLPNO-CCSD(T) variant, also shows outstanding accuracy for the anthracene series, with a maximum error of just 0.135 eV. A judicious choice of trial wavefunction for AFQMC, based on the AFQMC/U formalism described in ref. 53, is shown to be a promising tool for fast triplet energy screening, with all such calculations taking ≃2 hours of wall time on the Summit supercomputer.

However, when extending the data set to the BTD and BSeD series, which exhibit charge transfer characteristics and significant electron correlation effects, the accuracy of all TD-DFT functionals notably deteriorates as compared to the AFQMC reference values, with maximum deviations between 0.4 and 1.25 eV. DLPNO-CCSD(T) exhibits good agreement with AFQMC except for the case of Ph-BTD, where DLPNO-CCSD(T) overestimates the triplet energy by around 0.3 eV. While this discrepancy might be an artifact of unsuitable localization thresholds utilized in the default DLPNO implementation, our effort to use a more mild approximation proved intractable, highlighting the computational cost of the underlying CCSD(T) method. In the outlier case of Ph-BTD, the accuracy of our AFQMC prediction is experimentally validated by pairing with two sensitizers of known triplet energies, which provides further evidence that AFQMC can reliably produce quantitatively accurate relative spin state energetics for a wide variety of medium-sized organic molecules at an affordable computational cost. Additional low temperature phosphorescence measurements of the triplet energies of Ph-BTD provides further evidence for the enhanced predictive accuracy of AFQMC for the spin gaps of these organic systems. It is notable that in some cases Kohn–Sham B3LYP is surprisingly accurate, while in others it is wildly inaccurate; *e.g.*, for Ph-BTD, only KS-B3LYP correctly predicts exothermic TTA (along with AFQMC), but is off by 0.7 eV for the unsubstituted BTD.

Interestingly, a notable deviation between experiment and computational predictions was found for BTD and CN-BTD. This discrepancy would be ameliorated somewhat by including considerations of weak dimerization at low temperatures[see ESI†]. We note, however, that the possibility of dimerization is in conflict with both the very low concentration of BTD (≃ μM) in the phosphorescence experiments, and the speed at which the solution is cooled, and we do not consider this further. As the discrepancy for all electronic structure methods screened is additionally not due to basis set errors, and both AFQMC/C and DLPNO-CCSD(T) agree, these two cases warrant further investigation. Even with these notable outliers, AFQMC exhibits the lowest deviation (0.17 ± 0.07 eV) from available experiments (for DCA, DPA, and the BTD series) out of the computational methods screened, statistically equivalent to TD-B3LYP. But we note that in the important case of Ph-BTD, the newly discovered annihilator for TTA upconversion, TD-B3LYP overestimates the experimental triplet energy by some 0.3 eV.

A few comments are now in order, regarding the significance of our present discovery of the PtOEP/Ph-BTD upconverting system. While the reported anti-Stokes shift is not particularly remarkable compared with those of some existing blue or near UV emitting annihilators,^11,17,93–95^ our calculations suggest that Ph-BTD can achieve notably high energetic efficiency, *i.e.* minimal energetic loss during TTA upconversion. Moreover, we have demonstrated that derivatives of the BTD core are a new class of aromatic molecules that can participate in TTA upconversion, expanding the growing library of high energy annihilator structures. With appropriate sensitizer pairings, the BTD and BSeD derivatives investigated here are predicted to satisfy the thermodynamic requirements for photon upconversion, and to emit in the range of 2.8–3.9 eV. The computational methods validated in this work provide a platform for the rational design of novel upconverting systems, which can both screen for energetic efficiency and provide a link between chemical functionalization and tunable photophysical properties. With these tools as a guide, further investigations into unexplored corners of chemical space for the BTD and BSeD series are under way.

## 5 Conclusions

We have found that AFQMC is an *ab initio* methodology that is accurate in its predictions of triplet energies and scalable to realistic systems relevant to photophysical processes such as upconversion. We provide predictions for a variety of known and potential annihilators designed by adding substituent groups to anthracene, BTD, and BSeD frameworks. We find that triplet energies calculated from DFT and DLPNO-CCSD(T) methods show minimal deviations from the AFQMC values in the case of the anthracenes, with the B3LYP functional in the context of TD-DFT providing accuracy comparable to DLPNO-CCSD(T). Investigation of the BTD and BSeD series led to similar agreement among the theoretical approaches, with the notable exception of Ph-BTD, for which DLPNO-CCSD(T) and all TD-DFT methods overestimated the triplet energy by ≃0.35 to 0.60 eV compared to AFQMC. The AFQMC predictions are supported by experimental evidence of the occurrence of TET when Ph-BTD is coupled to a sensitizer with a larger triplet energy (PtOEP), but not when coupled to one with a smaller triplet energy (ZnTPP). Additionally, low-temperature phosphorescence measurements of Ph-BTD agree to within 0.04 ± 0.06 eV of AFQMC. Large deviations from phosphorescence values for BTD and CN-BTD for all methods were found, though the possibility of dimerization due to weak chalcogen bonding deserves further investigation.

Together with calculated S1 energies from the CAM-B3LYP/TD-DFT, which were shown to accurately predict a set of experimental measurements, the AFQMC triplet energies were used to investigate the energetic efficiency of TTA for all molecules. This led to the discovery of a novel annihilator, Ph-BTD, which when coupled to PtOEP emits upconverted blue light. This system exhibits an encouragingly small energy difference between twice T1 and S1, which results in less energetic loss through TTA, and thus high theoretical efficiency. More broadly, we have introduced a new class of upconverting annihilators which can be tuned *via* chemical functionalization to emit in the violet-UV regime.

This work echoes a previous study^96^ in highlighting the utility of computer simulations in the screening of TTA upconversion emitters for the rational design of upconverting materials. Yet crucially, the TD-DFT and DLPNO-CCSD(T) methods examined in this study would have led us to overlook the Ph-BTD/PtOEP pair, underscoring the importance of predictive accuracy on the level of around a tenth of an eV or less. In contrast to the other computational methods investigated here and, *e.g.*, in ref. 96, AFQMC is capable of providing this resolution for triplet energies, and thus will be a powerful tool for the design of upconverting annihilators.

## Conflicts of interest

There are no conflicts to declare.

## Supplementary Material

SC-012-D0SC03381B-s001

## References

[cit1] Ravetz B. D., Pun A. B., Churchill E. M., Congreve D. N., Rovis T., Campos L. M. (2019). Nature.

[cit2] Shaw M. H., Twilton J., MacMillan D. W. (2016). J. Org. Chem..

[cit3] Romero N. A., Nicewicz D. A. (2016). Chem. Rev..

[cit4] Zhou J., Liu Q., Feng W., Sun Y., Li F. (2014). Chem. Rev..

[cit5] Miyata K., Conrad-Burton F. S., Geyer F. L., Zhu X.-Y. (2019). Chem. Rev..

[cit6] Endo A., Ogasawara M., Takahashi A., Yokoyama D., Kato Y., Adachi C. (2009). Adv. Mater..

[cit7] Goldschmidt J. C., Fischer S. (2015). Adv. Opt. Mater..

[cit8] Atabaev T. S., Molkenova A. (2019). Frontiers of Materials Science.

[cit9] Sasaki Y., Oshikawa M., Bharmoria P., Kouno H., Hayashi-Takagi A., Sato M., Ajioka I., Yanai N., Kimizuka N. (2019). Angew. Chem., Int. Ed..

[cit10] Zhao J., Ji S., Guo H. (2011). RSC Adv..

[cit11] Haruki R., Sasaki Y., Masutani K., Yanai N., Kimizuka N. (2020). Chem. Commun..

[cit12] Nishimura N., Gray V., Allardice J. R., Zhang Z., Pershin A., Beljonne D., Rao A. (2019). ACS Mater. Lett..

[cit13] Börjesson K., Rudquist P., Gray V., Moth-Poulsen K. (2016). Nat. Commun..

[cit14] Gray V., Dzebo D., Lundin A., Alborzpour J., Abrahamsson M., Albinsson B., Moth-Poulsen K. (2015). J. Mater. Chem. C.

[cit15] Yanai N., Kozue M., Amemori S., Kabe R., Adachi C., Kimizuka N. (2016). J. Mater. Chem. C.

[cit16] Zhao W., Castellano F. N. (2006). J. Phys. Chem. A.

[cit17] Gray V., Xia P., Huang Z., Moses E., Fast A., Fishman D. A., Vullev V. I., Abrahamsson M., Moth-Poulsen K., Tang M. L. (2017). Chem. Sci..

[cit18] Singh-Rachford T. N., Castellano F. N. (2009). J. Phys. Chem. A.

[cit19] Huang L., Kakadiaris E., Vaneckova T., Huang K., Vaculovicova M., Han G. (2019). Biomaterials.

[cit20] Pun A. B., Campos L. M., Congreve D. N. (2019). J. Am. Chem. Soc..

[cit21] Ehrler B., Walker B. J., Böhm M. L., Wilson M. W., Vaynzof Y., Friend R. H., Greenham N. C. (2012). Nat. Commun..

[cit22] Völcker A., Adick H.-J., Schmidt R., Brauer H.-D. (1989). Chem. Phys. Lett..

[cit23] Fagnoni M. (2010). Angew. Chem..

[cit24] Hachmann J., Dorando J. J., Avilés M., Chan G. K.-L. (2007). J. Chem. Phys..

[cit25] Rosenberg M., Dahlstrand C., Kilsa K., Ottosson H. (2014). Chem. Rev..

[cit26] Bendikov M., Duong H. M., Starkey K., Houk K., Carter E. A., Wudl F. (2004). J. Am. Chem. Soc..

[cit27] Dreuw A., Head-Gordon M. (2005). Chem. Rev..

[cit28] Lee J., Head-Gordon M. (2019). J. Chem. Phys..

[cit29] Bernard Y. A., Shao Y., Krylov A. I. (2012). J. Chem. Phys..

[cit30] Slipchenko L. V., Krylov A. I. (2002). J. Chem. Phys..

[cit31] Gagliardi L., Truhlar D. G., Li Manni G., Carlson R. K., Hoyer C. E., Bao J. L. (2016). Acc. Chem. Res..

[cit32] Sharma P., Bernales V., Knecht S., Truhlar D. G., Gagliardi L. (2019). Chem. Sci..

[cit33] Zimmerman P. M. (2017). J. Phys. Chem. A.

[cit34] Yost S. R., Head-Gordon M. (2016). J. Chem. Phys..

[cit35] Lin Z., Van Voorhis T. (2019). J. Chem. Theory Comput..

[cit36] Liakos D. G., Guo Y., Neese F. (2019). J. Phys. Chem. A.

[cit37] Saitow M., Becker U., Riplinger C., Valeev E. F., Neese F. (2017). J. Chem. Phys..

[cit38] Sparta M., Neese F. (2014). Chem. Soc. Rev..

[cit39] Bao J. L., Sand A., Gagliardi L., Truhlar D. G. (2016). J. Chem. Theory Comput..

[cit40] Cui X., Charaf-Eddin A., Wang J., Le Guennic B., Zhao J., Jacquemin D. (2014). J. Org. Chem..

[cit41] Laurent A. D., Jacquemin D. (2013). Int. J. Quantum Chem..

[cit42] Xu K., Zhao J., Cui X., Ma J. (2015). J. Phys. Chem. A.

[cit43] Peach M. J., Williamson M. J., Tozer D. J. (2011). J. Chem. Theory Comput..

[cit44] Autschbach J., Srebro M. (2014). Acc. Chem. Res..

[cit45] Yanai T., Tew D. P., Handy N. C. (2004). Chem. Phys. Lett..

[cit46] Zhao Y., Truhlar D. G. (2008). Theor. Chem. Acc..

[cit47] Hirata S., Head-Gordon M. (1999). Chem. Phys. Lett..

[cit48] Brueckner C., Engels B. (2017). Chem. Phys..

[cit49] Moore B., Sun H., Govind N., Kowalski K., Autschbach J. (2015). J. Chem. Theory Comput..

[cit50] Gertsen A. S., Koerstz M., Mikkelsen K. V. (2018). Phys. Chem. Chem. Phys..

[cit51] Motta M., Zhang S. (2018). Wiley Interdiscip. Rev.: Comput. Mol. Sci..

[cit52] Zhang S. , Handbook of Materials Modeling: Methods: Theory and Modeling, 2018, pp. 1–27

[cit53] Shee J., Arthur E. J., Zhang S., Reichman D. R., Friesner R. A. (2019). J. Chem. Theory Comput..

[cit54] Shee J., Zhang S., Reichman D. R., Friesner R. A. (2017). J. Chem. Theory Comput..

[cit55] Shee J., Arthur E. J., Zhang S., Reichman D. R., Friesner R. A. (2018). J. Chem. Theor. Comput..

[cit56] Kent P., Annaberdiyev A., Benali A., Bennett M. C., Landinez Borda E. J., Doak P., Hao H., Jordan K. D., Krogel J. T., Kylänpää I. (2020). et al.. J. Chem. Phys..

[cit57] Malone F. D., Zhang S., Morales M. A. (2018). J. Chem. Theor. Comput..

[cit58] Singh-Rachford T. N., Islangulov R. R., Castellano F. N. (2008). J. Phys. Chem. A.

[cit59] Yu J., Ornelas J. L., Tang Y., Uddin M. A., Guo H., Yu S., Wang Y., Woo H. Y., Zhang S., Xing G., Guo X., Huang W. (2017). ACS Appl. Mater. Interfaces.

[cit60] Wang N., Chen Z., Wei W., Jiang Z. (2013). J. Am. Chem. Soc..

[cit61] Neto B. A., Carvalho P. H., Correa J. R. (2015). Acc. Chem. Res..

[cit62] Edelmann M. J., Raimundo J.-M., Utesch N. F., Diederich F., Boudon C., Gisselbrecht J.-P., Gross M. (2002). Helv. Chim. Acta.

[cit63] Zhang S., Carlson J., Gubernatis J. E. (1997). Phys. Rev. B: Condens. Matter Mater. Phys..

[cit64] Zhang S., Krakauer H. (2003). Phys. Rev. Lett..

[cit65] Ma F., Zhang S., Krakauer H. (2013). New J. Phys..

[cit66] Purwanto W., Zhang S., Krakauer H. (2009). J. Chem. Phys..

[cit67] Purwanto W., Al-Saidi W., Krakauer H., Zhang S. (2008). J. Chem. Phys..

[cit68] Lee J. , Malone F. D. and Morales M. A., arXiv preprint arXiv:2001.05109, 2020

[cit69] Landinez Borda E. J., Gomez J., Morales M. A. (2019). J. Chem. Phys..

[cit70] Shee J., Rudshteyn B., Arthur E. J., Zhang S., Reichman D. R., Friesner R. A. (2019). J. Chem. Theory Comput..

[cit71] Williams K. T., Yao Y., Li J., Chen L., Shi H., Motta M., Niu C., Ray U., Guo S., Anderson R. J. (2020). et al.. Phys. Rev. X.

[cit72] Sayfutyarova E. R., Hammes-Schiffer S. (2019). J. Chem. Theory Comput..

[cit73] Sun Q., Berkelbach T. C., Blunt N. S., Booth G. H., Guo S., Li Z., Liu J., McClain J. D., Sayfutyarova E. R., Sharma S., Wouters S., Chan G. K. (2018). Wiley Interdiscip. Rev.: Comput. Mol. Sci..

[cit74] Dunning Jr T. H. (1989). J. Chem. Phys..

[cit75] Kendall R. A., Dunning Jr T. H., Harrison R. J. (1992). J. Chem. Phys..

[cit76] Riplinger C., Pinski P., Becker U., Valeev E. F., Neese F. (2016). J. Chem. Phys..

[cit77] Guo Y., Riplinger C., Becker U., Liakos D. G., Minenkov Y., Cavallo L., Neese F. (2018). J. Chem. Phys..

[cit78] Neese F. (2012). Wiley Interdiscip. Rev.: Comput. Mol. Sci..

[cit79] Yang Y., Davidson E. R., Yang W. (2016). Proc. Natl. Acad. Sci. U. S. A..

[cit80] Rudshteyn B., Coskun D., Weber J. L., Arthur E. J., Zhang S., Reichman D. R., Friesner R. A., Shee J. (2020). J. Chem. Theory Comput..

[cit81] Fang Z., Lee Z., Peterson K. A., Dixon D. A. (2016). J. Chem. Theory Comput..

[cit82] Dixon A. R., Khuseynov D., Sanov A. (2015). J. Chem. Phys..

[cit83] Darmanyan A. (1984). Chem. Phys. Lett..

[cit84] Ieuji R., Goushi K., Adachi C. (2019). Nat. Commun..

[cit85] Warren J. D., Lee V. J., Angier R. B. (1979). J. Heterocycl. Chem..

[cit86] Mancilha F. S., DaSilveira Neto B. A., Lopes A. S., Moreira Jr P. F., Quina F. H., Gonçalves R. S., Dupont J. (2006). Eur. J. Org. Chem..

[cit87] Lopata K., Reslan R., Kowalska M., Neuhauser D., Govind N., Kowalski K. (2011). J. Chem. Theory Comput..

[cit88] Baluschev S., Yakutkin V., Wegner G., Minch B., Miteva T., Nelles G., Yasuda A. (2007). J. Appl. Phys..

[cit89] Walters V. A., de Paula J. C., Jackson B., Nutaitis C., Hall K., Lind J., Cardozo K., Chandran K., Raible D., Phillips C. M. (1995). J. Phys. Chem..

[cit90] Cheng Y. Y., Fuckel B., Khoury T., Clady R. G., Ekins-Daukes N., Crossley M. J., Schmidt T. W. (2011). J. Phys. Chem. A.

[cit91] Isokuortti J., Allu S. R., Efimov A., Vuorimaa-Laukkanen E., Tkachenko N. V., Vinogradov S. A., Laaksonen T., Durandin N. A. (2019). J. Phys. Chem. Lett..

[cit92] Tsuzuki S., Sato N. (2013). J. Phys. Chem. B.

[cit93] Gao C., Zhang B., Hall C. R., Li L., Chen Y., Zeng Y., Smith T. A., Wong W. W. (2020). Phys. Chem. Chem. Phys..

[cit94] Deng F., Blumhoff J., Castellano F. N. (2013). J. Phys. Chem. A.

[cit95] Duan P., Yanai N., Nagatomi H., Kimizuka N. (2015). J. Am. Chem. Soc..

[cit96] Wang X., Tom R., Liu X., Congreve D., Marom N. (2020). J. Mater. Chem. C.

