# Supplementary material for: In silico prediction of annihilators for triplet–triplet annihilation upconversion via auxiliary-field quantum Monte Carlo
Source: Chem Sci. 2020 Nov 17;12(3):1068–79. doi: 10.1039/d0sc03381b (PMC8179011; doi:10.1039/d0sc03381b)
Supplement: SC-012-D0SC03381B-s001 [file SC-012-D0SC03381B-s001.pdf]

Electronic Supplementary Material (ESI) for Chemical Science.
This journal is © The Royal Society of Chemistry 2020

In-Silico Prediction of Annihilators for Triplet-Triplet Annihilation Upconversion via Auxiliary-Field Quantum Monte Carlo

John L. Weber,[†] Emily M. Churchill,[†] Steffen Jockusch,[†] Evan J. Arthur,[‡] Andrew B. Pun,[†] Shiwei Zhang,[¶,§] Richard A. Friesner,[†] Luis M. Campos,[†] David R. Reichman,[†] and James Shee[*,†]

†*Department of Chemistry, Columbia University, 3000 Broadway, New York, NY, 10027*

‡*Schrodinger Inc., 120 West 45th Street, New York, NY, 10036*

¶*Center for Computational Quantum Physics, Flatiron Institute, 162 5th Avenue, New York, NY 10010*

§*Department of Physics, College of William and Mary, Williamsburg, VA 23187*

E-mail: js4564@columbia.edu

Supporting Information Available

S1 Experimental Methods

All starting materials were obtained from commercial chemical sources, including Fisher Scientific, TCI Chemical, and Strem Chemicals. BTD (ACROS Organics), ZnTPP (Fisher Scientific), and PtOEP (Sigma-Aldrich) were purchased and used without further purification. Materials Ph-BTD,[1] MeO-BTD,[2,3] and CN-BTD[4] were synthesized according to previously reported procedures.

NMR spectra were collected on a Bruker 500 MHz spectrometer at ambient temperature. UV-Vis absorption spectra were collected by a Technologies Cary 60 UV-Vis Spectrophotometer. Steady-state photoluminescence spectra were collected by an Ocean Optics QEPro Spectrometer.

S1.1 TTA Upconversion Analysis

Solutions were made with 1x10^{-}5 M sensitizer and 1x10^{-}3 M annihilator in degassed anhydrous toluene. Solutions for each sensitizer-annihilator pair were made in a nitrogen glovebox, sealed, and removed from the glovebox for upconversion photoluminescence study.

Normalized emission and absorption spectra of sensitizers PtOEP and ZnTPP are seen in figures S1 and S2, where dashed lines denote absorption and solid lines denote emission upon excitation at 365 nm.

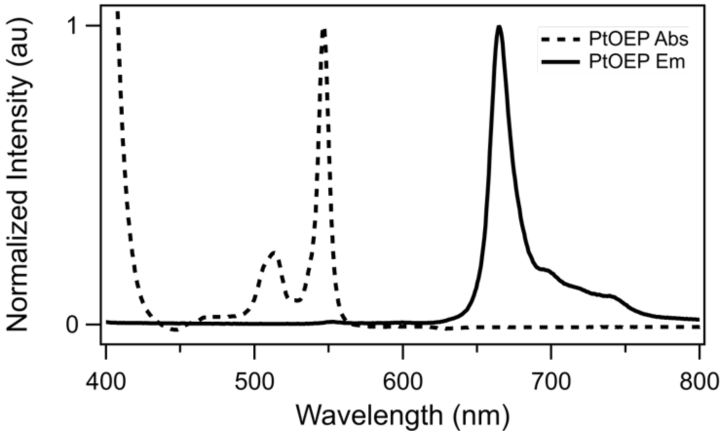

Figure S1: Absorption (dashed) and photoluminescence (solid) of PtOEP in toluene.

Normalized absorption spectra of sensitizers PtOEP and ZnTPP and emission of solution mixture of the sensitizers paired with BTD are seen in figures S5 and S4, where dashed lines denote the absorption of the sensitizer and solid lines denote the emission upon excitation at 532 nm.

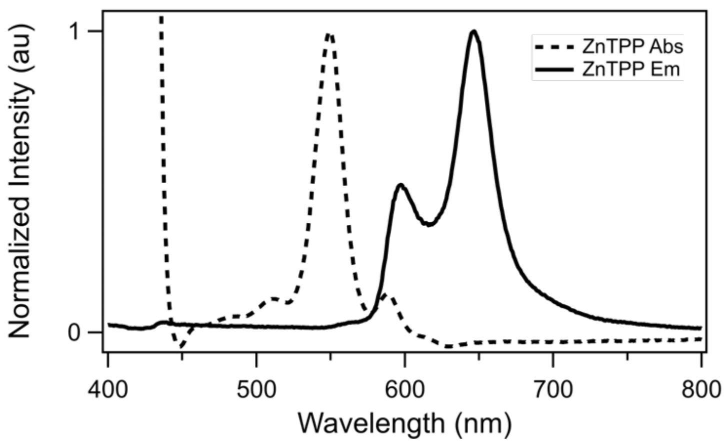

Figure S2: Absorption (dashed) and photoluminescence (solid) of ZnTPP in toluene.

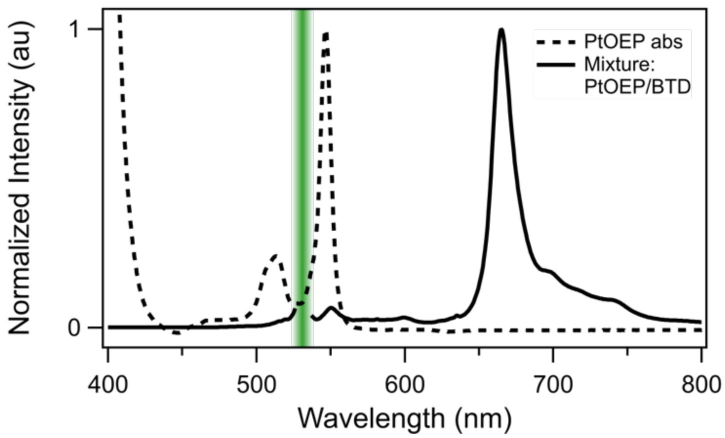

Figure S3: Absorption (dashed) spectrum of PtOEP, and photoluminescence (solid) of the BTD/PtOEP pair in toluene with excitation at 532 nm (green line). The emission of the mixture matches that of PtOEP (Figure S1), supporting that there is no TET from PtOEP to BTD in solution.

S1.2 Phosphorescence Measurements

Time-resolved phosphorescence measurements for BTD and CN-BTD were recorded on a Fluorolog-3P fluorometer (HORIBA Jobin Yvon). Sample solutions in 3-mm quartz tubes (inner diameter) were frozen in a quartz liquid nitrogen Dewar and excited with a pulsed xenon lamp. Luminescence signal detection was delayed 10 ms (for BTD) or 1 ms (for CN-BTD) after the light pulse and collected for 20 ms (BTD) or 1 ms (CN-BTD). Phosphorescence lifetimes (T_p) at 77 K for BTD and CN-BTD were measured by multichannel scaling on an OB920 spectrometer (Edinburgh Analytical Instruments) in conjunction with a pulsed xenon lamp. Time-resolved phosphorescence measurements of MeO-BTD and Ph-

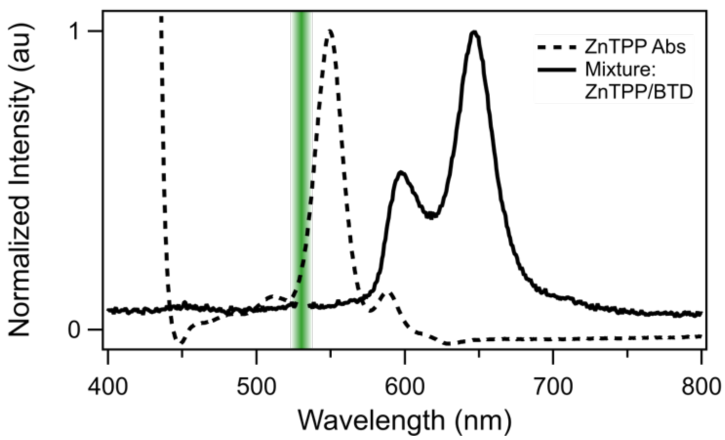

Figure S4: Absorption (dashed) spectrum of ZnTPP, and photoluminescence (solid) of the BTD/ZnTPP pair in toluene with excitation at 532 nm (green line). The emission of the mixture matches that of ZnTPP, (Figure S2), supporting that there is no TET from ZnTPP to BTD in solution.

BTD were measured in frozen methylcyclohexane/iodomethane (2:1, v/v) in 3 mm quartz tubes (inner diameter) at 77 K in a optical liquid N_2 quartz dewar. Iodomethane was added to increase intersystem crossing into the triplet state. The frozen samples inside the quartz dewar were excited with a pulsed Spectra Physics GCR-150-30 Nd:YAG laser (355 nm, ca. 1 mJ per pulse, 5 ns pulse length). The time-resolved phosphorescence spectra at 100 μs (for MeO-BTD) or 20 μs (for Ph-BTD) after pulsed excitation and a gate width of 500 μs (MeO-BTD) or 1 ms (Ph-BTD) were recorded on an Acton Spectrograph (SpectraPro-2150) in conjunction with an intensified CCD detector (PI-MAX from Princeton Instruments) with fiber optics attachment. The triplet energies (E_T) were determined from the highest energy peaks of the phosphorescence spectra of BTD, CN-BTD and MeO-BTD. Because the phosphorescence spectrum of Ph-BTD did not show resolved vibrational peaks, the triplet energy was estimated to have a value between 1.85 eV (phosphorescence maximum) and 1.99 eV (wavelength of $\frac{1}{2}$ intensity of the phosphorescence peak).

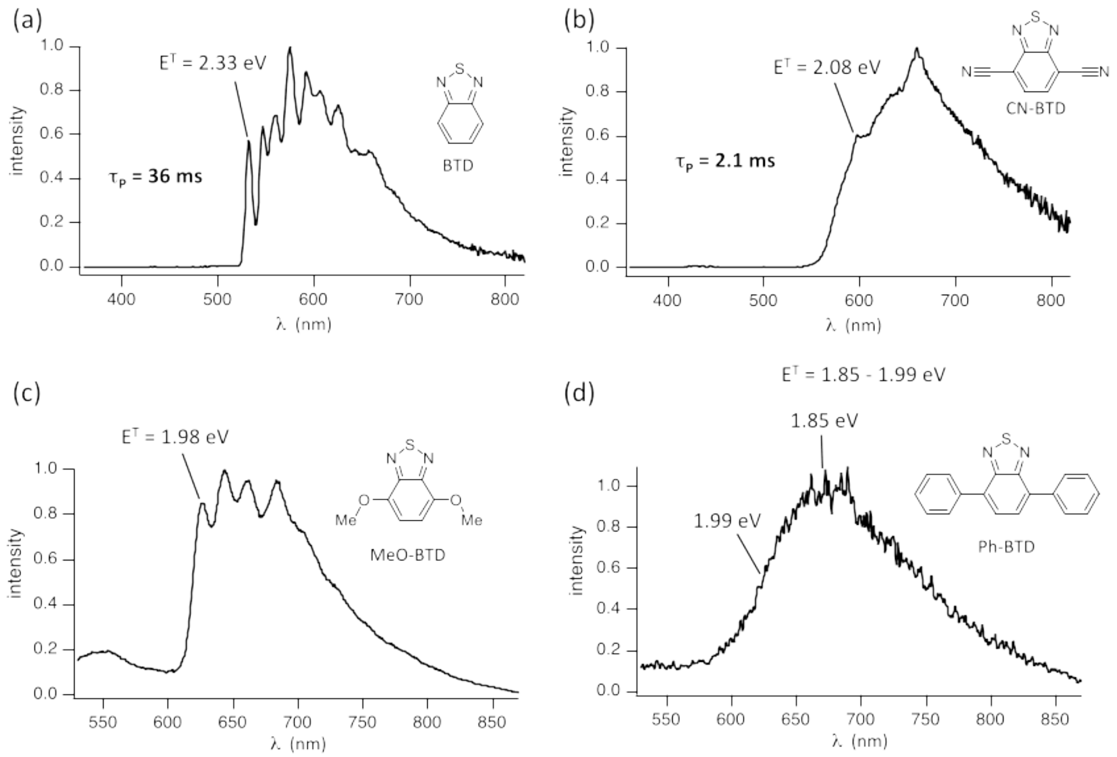

Figure S5: Time-resolved phosphorescence spectra of BTD derivatives in methylcyclohexane (a, b) or methylcyclohexane / iodomethane mixture (2:1, v/v) (c, d) at 77 K. λex = 340 nm (a, b) or λex = 355 nm (c, d). Detection window after light pulse: 10 - 30 ms (a), 1 - 6 ms (b), 100 - 600 μs (c), 20 - 1020 μs (d).

S2 Additional Calculation Details

All AFQMC calculations in this work use the same procedures and parameters (e.g. Cholesky decomposed two-electron integrals, time-step size, precision for floating-point operations, orthonormalization interval, etc.) detailed in Ref. 5, with walker populations of around 1200.

We additionally explored the effects of extrapolating our triplet energies, as computed with ph-AFQMC in the cc-pVTZ (TZ) basis, to the complete basis set (CBS) limit using DLPNO-CCSD(T) values in TZ and QZ (see Ref. 6 for details of this protocol). For the subset of molecules in Section 3.1.1 we find that with DLPNO-CCSD(T) the difference between the TZ result and our extrapolated estimate of the CBS limit is never larger than 0.06 eV. Given that our QMC error bars are larger than this, further extrapolation of the AFQMC/U results appears to be unnecessary for screening, and the following results reflect

use of the TZ basis. This is consistent with previous CC[7] and DFT[8] studies showing TZ basis sets to be sufficient for screening T1 excitation energies of organic chromophores. This was not the case for the BTD series, and we therefore make use of CBS numbers in the main text. Species with no CBS values indicate that we were unable to converge QZ DLPNO-CCSD(T) calculations in <1 week computational wall time.

S2.1 Triplet energies

Below are our calculated adiabatic T1 energies for all systems involved.

Table S1: A comparison in eV of AFQMC/U, DLPNO-CCSD(T), KS-DFT, and TD-DFT results for the T1 energies of anthracene derivatives. Parentheses denote statistical error; the value to the right of "/" denotes the value extrapolated to the basis set limit.

	KS-B3LYP	B3LYP	CAM-B3LYP	M062X	DLPNO-CCSD(T)(TZ/CBS)	AFQMC(TZ/CBS)
DCA	1.50	1.757	1.825	1.956	1.76/1.80	1.74(7)/1.80(7)
DPA	1.66	1.940	2.023	2.123	2.02/2.04	1.89(11)/1.93(10)
CF3	1.66	1.875	1.943	2.075	2.02/2.03	2.09(11)/2.14(10)
CN-updn-Me	1.28	1.688	1.766	1.895	1.74/1.76	1.70(12)/1.74(11)
CN-diag-Me	1.42	1.790	1.865	1.996	1.79/1.82	1.80(11)/1.86(10)
DMA	1.54	1.951	2.025	2.148	1.91/1.92	1.81(7)/1.84(7)
OMe	1.60	2.017	2.093	2.207	1.94/1.94	1.87(10)/1.90(9)
Ac	1.41	1.688	1.749	1.537	1.68	1.68(8)
Ac-CN1	1.38	1.649	1.718	1.502	1.67	1.56(6)
Ac-CN2E	1.36	1.611	1.686	1.464	1.65	1.64(7)
Ac-CN2Z	1.37	1.622	1.697	1.480	1.67	1.67(7)

Table S2: A comparison in eV of AFQMC/CAS, DLPNO-CCSD(T), KS-DFT, and TD-DFT results for T1 of a set of substituted BTD and BSeD compounds. Parentheses denote statistical error; the value to the right of "/" denotes the value extrapolated to the basis set limit.

Species	KS-B3LYP	B3LYP	CAM-B3LYP	M062X	DLPNO-CCSD(T)	AFQMC
BTD	3.11	2.594	2.647	2.828	2.87/2.67	2.85(6)/2.66(7)
CN-BTD	1.99	2.299	2.358	2.528	2.28/2.37	2.33(7)/2.43(6)
MeO-BTD	1.89	2.148	2.350	2.509	2.17/2.22	2.18(7)/2.20(6)
Ph-BTD	1.78	2.154	2.289	2.437	2.07/2.13	1.77(6)/1.81(6)
BSeD	2.81	2.339	2.381	2.558	2.60/2.84	2.65(4)/2.89(3)
CN-BSeD	1.83	2.143	2.188	2.357	2.13/2.36	2.25(7)/2.50(7)
MeO-BSeD	1.66	1.913	2.098	2.246	1.98	1.87(5)
Ph-BSeD	1.62	1.961	3.352	2.233	1.92/1.96	2.11(10)/2.17(9)

Table S3: Mean Absolute Deviations for all experimental values (DCA, DPA, BTD, CN-BTD, MeO-BTD, Ph-BTD

Species	KS-B3LYP	B3LYP	CAM-B3LYP	M062X	DLPNO-CCSD(T)(TZ/CBS)	AFQMC(TZ/CBS)
MAD	0.249	0.171	0.257	0.405	0.216	0.170(73)

S2.2 Solvent and vibrational corrections

For a small subset of anthracene derivatives, the effect of vibrational zero-point energies and solvent corrections were evaluated. Solvent corrections were included using an implicit solvent model dielectric (CPCM) for toluene at the DFT level, and the corrected ST gaps are given in Table S5. As neither ZPE nor solvent correction resulted in an energetic shift greater than the statistical error of AFQMC, we neglect vibrational and solvent effects in the main manuscript, and expect (and find) that while the reported energies are gas phase, they accurately reflect solvated energetics in a nonpolar solvent such as toluene.

The vibrational corrections were estimated from numerical vibrational frequency calculations with an increment of 0.001 Bohr. Zero-point energies and thermal vibrational corrections are scaled by a factor obtained from the NIST computational chemistry database (0.965 for B3LYP/cc-pVTZ)[9] and added to the resulting energies. Corrections due to solvation effects were obtained from geometries and vibrational frequencies computed within the conductor-like polarizable continuum model (CPCM) to estimate the effects of toluene solvent. The DLPNO-CCSD(T) and AFQMC/U values are obtained by first calculating the S0 - T1 energy difference in the gas-phase; energetic corrections to this gap due to ZPEs and thermal occupation of vibrational states, computed in the gas-phase, are scaled and then added. Finally, a solvent correction is obtained via DFT as the difference between the vibrationally corrected ST gaps in toluene, as represented by the CPCM model, and in gas-phase.

Table S4: Dielectric corrections to the gas-phase energy to account for solvent (toluene) effects, within the CPCM formalism. All corrections were found at the B3LYP/TZ level, using ORCA. Note that tthe solvation correction is minimal in this low-dielectric solvation model, as expected, aside from the MeO-substituted diazoles, which exhibit the strongest charge-transfer due to the electron donating character of MeO; the corrections were -0.15 and -0.13 eV for MeO-BTD and MeO-BSeD, respectively.

Species	Solvation Correction
DMA	-0.036
DCA	0.063
OMe	0.012
CN-1,5-Me	-0.066
CN-2,6-Me	-0.008
CF3	0.028
DPA	-0.005
Ac	0.000
Ac-CN1	-0.006
Ac-CN2E	-0.013
Ac-CN2Z	0.005
BTD	-0.043
CN-BTD	0.032
MeO-BTD	-0.148
Ph-BTD	-0.003
BSeD	-0.046
CN-BSeD	0.042
MeO-BSeD	-0.128
Ph-BSeD	0.000

Table S5: Triplet energies including both vibrational and solvent (toluene) corrections within the CPCM formalism.

Species	KS-B3LYP	DLPNO-CCSD(T)	AFQMC/U
DMA	1.54	1.77	1.69
DCA	1.50	1.75	1.75
OMe	1.60	1.85	1.81
CN-1,5-Me	1.28	1.58	1.56
CN-2,6-Me	1.42	1.70	1.73
CF3	1.66	1.95	2.05
DPA	1.66	1.91	1.82

S2.2.1 Dimerization Energies

Due to prior computational evidence for strong chalcogen bonding in anti-square thiadiazole dimers,[10] we evaluated the electronic bonding energy for each pair. The bonding energies, evaluated at the KS-DFT ωB97X-V/cc-pVTZ level of theory, are shown in Table S6, in

addition to DLPNO-CCSD(T)/cc-pVTZ estimations of the triplet energy for each dimer. Each species electronically favors the dimer, with CN-BTD and BTD having the strongest and second strongest bonding energies, respectively. Moreover, the computational triplet energies agree with the experimental phosphorescence results, suggesting that the low temperature phosphorescence measurements may reflect dimerized BTD and CN-BTD species. To further evaluate the likelihood of dimerization, we calculated the dimerization free energies at a range of temperatures (Table S7). For a concentrated solution at low temperatures, the dimer is favored in both BTD and CN-BTD at ratios of 1000:1 and 1000,000,000:1, respectively. However, the likelihood of dimerization at low concentrations such as those used in the phosphorescence measurements ($\simeq \mu$M) is expected to be low.

Table S6: Electronic bond energies (eV) for the anti-square dimer (at the wB97X-V/cc-pVTZ level of theory), along with a comparison of triplet energies (eV) for the dimer(calculated by DLPNO-CCSD(T)) and monomer (AFQMC) vs experiment. All species exhibit chalcogen bonding, with CN-BTD and BTD exhibiting the highest and second highest bonding strengths, respectively. The phosphorescence for Ph-BTD lacked the fine structure necessary to specify the 0-0 transition energy, and so we report two estimates, one based on the phosphorescence maximum, and one (in parentheses) determined from the 1/2 intensity of the phosphorescence maximum. Triplet energies for the dimers, calculated with DLPNO-CCSD(T), agree with experiment for BTD and CN-BTD, within 0.04 and 0.1 eV, respectively.

	BTD	CN-BTD	MeO-BTD	Ph-BTD
Electronic Bond energy	0.179	0.279	0.125	0.124
Experimental T1 energy	2.33	2.07	1.98	1.85(1.99)
Dimer T1 energy (DLPNO-CCSD(T)/TZ)	2.42	2.28	2.22	2.10
Monomer gap (AFQMC/CBS)	2.62(6)	2.46(7)	2.09(7)	1.81(6)

Table S7: Dimerization free energies evaluated using KS-DFT at the B3LYP/cc-pVTZ level. Note that a negative free energy favors the formation of a dimer; all species are monomers at room temperature, but BTD and CN-BTD transition to a favored dimer state at low temperatures. Inspection of the geometries reveal in-plane dimerization for all but Ph-BTD, which is sterically limited to out of plane, likely resulting in the higher observed dimerization free energies.

Temperature (K)	BTD	CN-BTD	MeO-BTD	Ph-BTD
77	-0.040	-0.139	0.030	0.132
100	0.002	-0.096	0.076	0.199
150	0.097	-0.001	0.176	0.355
200	0.192	0.096	0.277	0.520
250	0.289	0.194	0.380	0.693
300	0.383	0.325	0.515	0.900

S2.3 Excited State Tuning

Here we attempt to rationalize shifts in the S0-S1 transition energies (Table S8) between functionalized chromophores based on intuitive concepts within chemistry. We find that two factors, that of π-system extension and a combination of electron-withdrawing and electron-rich substituents, can be used in tandem to lower the HOMO-LUMO gap.

Table S8: CAM-B3LYP TD-DFT results for S1 energies in eV.

Species	S1 (TD-DFT)
DPA	3.136
DMA	3.101
OMe	3.059
CF3	3.033
CN-2,6-Me	2.926
DCA	2.922
CN-1,5-Me	2.790
Ac	2.88
Ac-CN1	2.82
Ac-CN2E	2.78
Ac-CN2Z	2.77

Species	S1 (TD-DFT)
BTD	3.86
CN-BTD	3.64
MeO-BTD	3.05
Ph-BTD	3.04
BSeD	3.57
CN-BSeD	3.41
MeO-BSeD	2.78
Ph-BSeD	2.85

S2.3.1 π-extension

Extending the π system should result in lowering T1 and S1, thus substantially lowering the 2T1-S1 gap. In the series of anthracenes, it can be seen that the amount of S1 lowering from the parent system can be directly correlated to the extent of π-extension within the functional groups. Interestingly, Me groups contribute significantly to π-extension through hyper-conjugation, as seen in the HOMO-LUMO plots of DMA:

Whereas these particle in a box arguments are helpful in predicting S1, T1 is much more dependent on the exact nature of the functional group. We are currently unable to resolve any correlations that could be used as a design principle for T1 in the anthracenes, and in some cases T1 actually follows opposite trends from S1 (i.e. DMA $\rightarrow$ OMe $\rightarrow$ CF3). This, however, underlines the importance of accurate computational methods such as AFQMC.

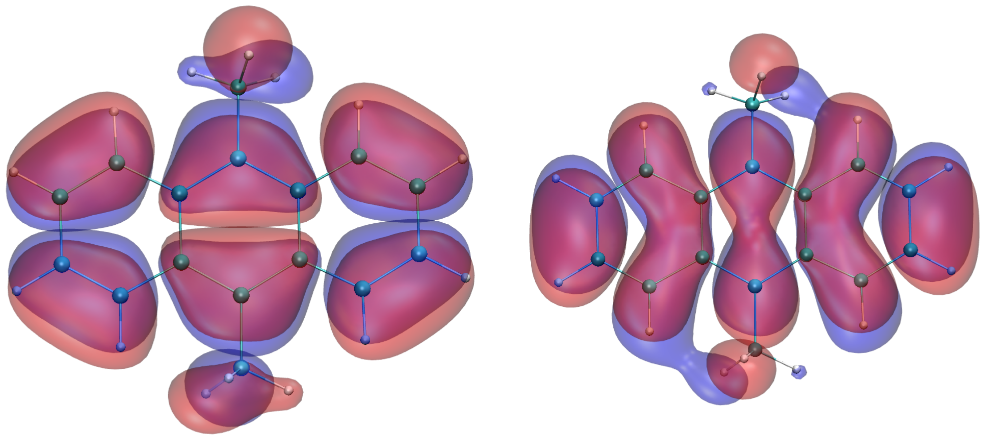

Figure S6: HOMO(left) and LUMO (right) orbital plots for DMA. Note the extension of the π-system into the Methyl groups.

S2.3.2 Donor-Acceptor Character

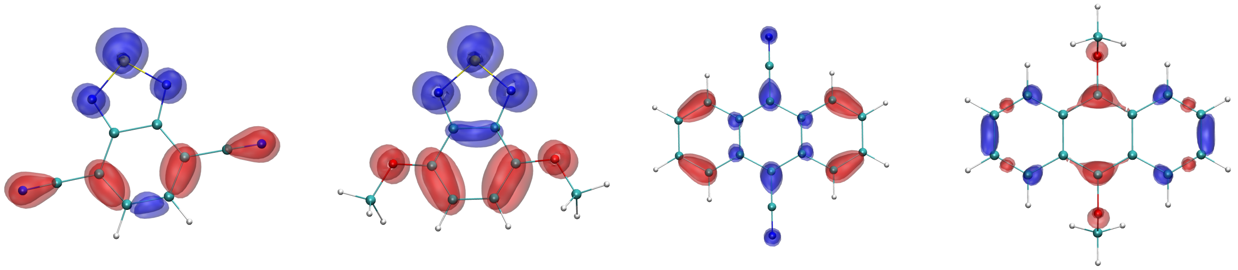

Figure S7: Difference-density plots for the S0-S1 transition for CN and MeO substituted BTD and anthracene. A red surface denotes electron loss upon excitation, while the blue is electron density gained. Note the significant charge transfer in both BTD cases from the functional groups to the sulfur ring, regardless of electron withdrawing (CN) or donating (OMe) character; withdrawal of electrons from CN likely raises the excited state energy in comparison to OMe, even with similar HOMO-LUMO π-extension. In comparison, DCA and OMe-anthracene show the opposite trend, while the difference density plot shows electron withdrawing to the CN and electron donation from the OMe.

π-system extension can have neutral, donating, or withdrawing character, and excitations can involve varying degrees and directions of charge-transfer, which can in principle be harnessed to lower the ST gap via judicious choice of solvent polarity.[11] This may also be a factor in increasing the energy of certain excitation states by pulling electrons away from highly electronegative groups within certain geometries. In a similar fashion, it is well known that conjugation of an donor-acceptor pair can lead to a large decrease in the band gap.[12]

We can therefore use the relative electron withdrawing/donating characters of the parent compound and functional groups to predict the extent of S1 lowering. The BTD series illustrates this well. BTD is a highly electron-poor system used frequently in donor acceptor paradigms, and so electron donating groups such as OMe lower the gap significantly more than electron withdrawing groups such as CN, even though OMe is arguably less effective at extending the π-system than CN (Figure S7).

S2.4 Singlet Excited states using AFQMC

Ideally, one would obtain both S1 and T1 from a consistent level of theory. In the case of calculating S1 for a molecule with a singlet ground state, fixing the spin cannot be used to orthogonalize the trial wave-function against the ground state, as it can for the triplet. In the case of anthracene, we fix the symmetry of the wavefunction to be B2u, that of the first bright excited singlet state. For a general non-symmetric molecule, one can use state-averaging techniques to obtain orthogonal CASSCF trial wavefunctions for an arbitrary number of excited states. We include all π orbitals in the active space, and use the same random number seeds for S0, S1, and T1 calculations to accelerate the convergence of the energy gaps, as in correlated sampling (procedure outlined in Ref. 13). Since correlated sampling attains maximum efficiency when the same geometries are used, we compute the vertical excitation energies, and thus compare to experiments corrected with a geometry reorganization energy calculated with DLPNO-CCSD(T) (from which, given our results for the anthracene derivatives, we expect reasonable accuracy). Indeed, in Table S9 we report vertical excitation energies within 0.05 eV accuracy versus experiment.

Table S9: AFQMC results for both S1 and T1 of Anthracene using Correlated Sampling

State	AFQMC/CAS	Experiment [14]
S1	3.65(4)	3.60
T1	2.27(1)	2.23[a]

[a] Corrected from experimental adiabatic energy using DLPNO-CCSD(T) reorganization energy

S2.5 Functionalized Tetracene structures

We have used a set of structures based on functionalizing tetracene with cyano groups in order to validate the use of CAM-B3LYP TDDFT to obtain S1 energies against experiment (see Table S8). The structures for these compounds can be seen below:

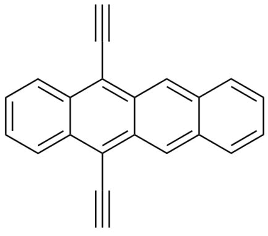

CN0

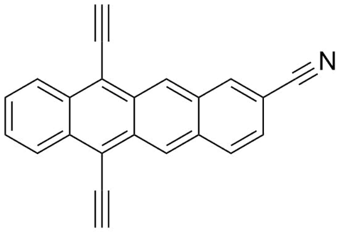

CN1

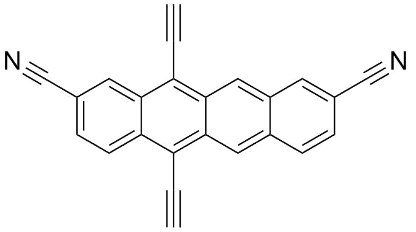

CN2E_cis

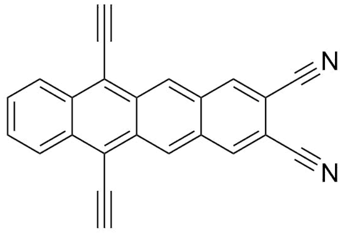

CN2T

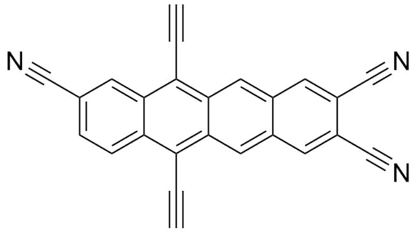

CN3

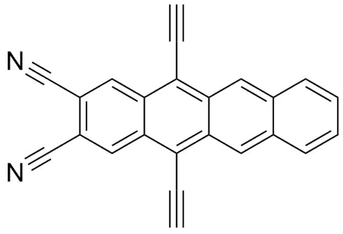

CN2H

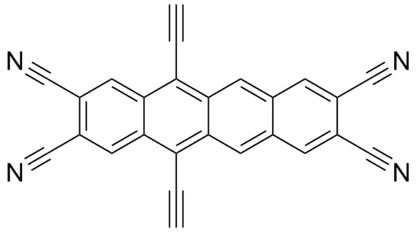

CN4

References

(1) Jiménez-Urias, A.; Lugo-Aranda, A. Z.; Miranda-Olvera, M.; Farfán, N.; Santillan, R.; Arcos-Ramos, R.; del Pilar Carreón-Castro, M. Synthesis and characterization of dumbbell-like BTD-based derivatives to engineer organic building blocks in solid-state. *Journal of Molecular Structure* **2018**, *1153*, 34 – 41.

(2) Lei, T.; Zhou, Y.; Cheng, C.-Y.; Cao, Y.; Peng, Y.; Bian, J.; Pei, J. Aceno[2,1,3]thiadiazoles for Field-Effect Transistors: Synthesis and Crystal Packing. *Organic Letters* **2011**, *13*, 2642–2645, PMID: 21510693.

(3) Zeng, S.; Yin, L.; Ji, C.; Jiang, X.; Li, K.; Li, Y.; Wang, Y. D—A—D type benzothiadiazole–triphenylamine based small molecules containing cyano on the -bridge for solution-processed organic solar cells with high open-circuit voltage. *Chem. Commun.* **2012**, *48*, 10627–10629.

(4) Zhao, Y.; Han, X.; Yu, F.; Wei, D.; Cheng, Q.; Meng, X.; Ding, J.; Hou, H. Direct Conversion of Benzothiadiazole to Benzimidazole: NewBenzimidazole-Derived Metal–Organic Frameworks with Adjustable Honeycomb-Like Cavities. *Chemistry – A European Journal* **2019**, *25*, 5246–5250.

(5) Shee, J.; Arthur, E. J.; Zhang, S.; Reichman, D. R.; Friesner, R. A. Singlet Triplet Energy Gaps of Organic Biradicals and Polyacenes with Auxiliary-Field Quantum Monte Carlo. *J. Chem. Theory Comput.* **2019**, *15*, 4924–4932, PMID: 31381324.

(6) Shee, J.; Rudshteyn, B.; Arthur, E. J.; Zhang, S.; Reichman, D. R.; Friesner, R. A. On Achieving High Accuracy in Quantum Chemical Calculations of 3d Transition Metal-containing Systems: A Comparison of Auxiliary-Field Quantum Monte Carlo with Coupled Cluster, Density Functional Theory, and Experiment for Diatomic Molecules. *J. Chem. Theory Comput.* **2019**,

(7) Schreiber, M.; Silva-Junior, M. R.; Sauer, S. P.; Thiel, W. Benchmarks for electronically excited states: CASPT2, CC2, CCSD, and CC3. *The Journal of chemical physics* **2008**, *128*, 134110.

(8) Goerigk, L.; Grimme, S. Assessment of TD-DFT methods and of various spin scaled CIS (D) and CC2 versions for the treatment of low-lying valence excitations of large organic dyes. *The Journal of Chemical Physics* **2010**, *132*, 184103.

(9) Johnson III, R. J. NIST Computational Chemistry Comparison and Benchmark Database. http://cccbdb.nist.gov/, NIST Standard Reference Database Number 101, Release 17b, September 2015.

(10) Tsuzuki, S.; Sato, N. Origin of Attraction in Chalgogen–Nitrogen Interaction of 1, 2, 5-Chalcogenadiazole Dimers. *The Journal of Physical Chemistry B* **2013**, *117*, 6849–6855.

(11) Sun, H.; Hu, Z.; Zhong, C.; Chen, X.; Sun, Z.; Bredas, J.-L. Impact of dielectric constant on the singlet–triplet gap in thermally activated delayed fluorescence materials. *The journal of physical chemistry letters* **2017**, *8*, 2393–2398.

(12) Hashemi, D.; Ma, X.; Ansari, R.; Kim, J.; Kieffer, J. Design principles for the energy level tuning in donor/acceptor conjugated polymers. *Physical Chemistry Chemical Physics* **2019**, *21*, 789–799.

(13) Shee, J.; Zhang, S.; Reichman, D. R.; Friesner, R. A. Chemical Transformations Approaching Chemical Accuracy via Correlated Sampling in Auxiliary-Field Quantum Monte Carlo. *J. Chem. Theory Comput.* **2017**, *13*, 2667–2680.

(14) Yang, Y.; Davidson, E. R.; Yang, W. Nature of ground and electronic excited states of higher acenes. *Proc. Natl. Acad. Sci.* **2016**, *113*, E5098–E5107.
